# XXVIII^e^ Actualités du Pharo. La santé au travail, entre famille et entreprise : quels enjeux pour les pays du Sud ? 4-6 octobre 2023, Marseille, France: Organisateur : GISPE (Groupe d’intervention en santé publique et épidémiologie), 82 boulevard Tellène, 13008 Marseille, France

**DOI:** 10.48327/mtsi.v4i2.2024.526

**Published:** 2024-06-07

**Authors:** Jean-Paul BOUTIN

**Affiliations:** GISPE, 82 boulevard Tellène, 13008 Marseille, France, www.gispe.org

## Éditorial

La santé au travail est un droit pour tous les travailleurs, mais reste un défi pour beaucoup d’entre eux comme pour beaucoup de pays. Pour cette 28^e^ édition des Actualités du Pharo le GISPE (Groupe d'intervention en santé publique et en épidémiologie) a fait le choix périlleux d'aborder un pan entier de la santé humaine qui est éminemment politique, mais reste un parent pauvre des programmes de santé. Même dans les régions de la planète réputées développées, là où les programmes de santé humaine ne se comptent plus, et où de réels succès sont obtenus dans de nombreux domaines, la santé au travail reste en retrait en termes de choix politiques, financiers, organisationnels, de considération, d'intérêt etc. Nul ne pourra se mettre en avant quel que soit son pays d'exercice devant un tel sujet ! Quel constat faire alors dans les pays du Sud où le contexte est encore plus défavorable, avec un secteur du travail informel important rendant très difficile l'application d'une politique publique, mais aussi où le secteur assuranciel peine à se généraliser. Le sujet est tellement vaste et divers qu'il convenait de commencer ces Actualités par une remise en contexte historique ou, comme le dit la Professeure J. Rainhorn « un coup dœil dans le rétroviseur pour contribuer à comprendre les défis contemporains » d'un champ de la santé communautaire malmené au Nord comme au Sud. Certes des efforts importants sont menés, et d'abord dans les organisations internationales, mais les résultats sont encore minces, d'autant que le sort de la santé des travailleurs et des travailleuses est extrêmement lié au développement préalable de toutes les composantes d'une politique sanitaire polyvalente : densité des soignants et des structures de soins, qualité des soins, possibilités de diagnostic, législation, assurances sociales, adhésion du monde de l'emploi, budget, surveillance épidémiologique, capacité d'enquêtes et d'expertise en milieu de travail, etc.

L'immensité de la tâche ne doit pas nous décourager et cette rencontre entre tropicalistes et acteurs de la santé au travail sera une pierre apportée à l'édifice à bâtir.

**Figure 1:**
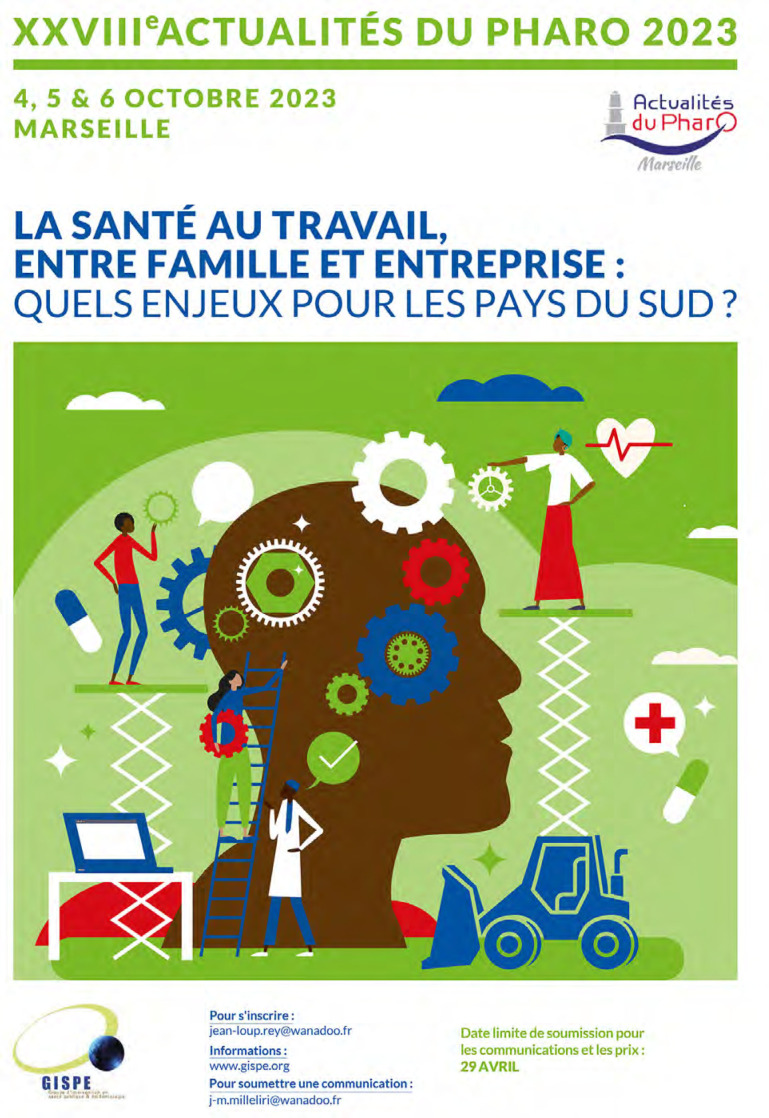
Affiche des XXVIII^e^ Actualités du Pharo. La santé au travail, entre famille et entreprise : quels enjeux pour les pays du Sud ? Poster of the XXVIII^th^ Actualités du Pharo. Health at work, between family and company: what are the issues for Southern countries?

