# Supplementary material for: XXVIIIth Actualités du Pharo. Health at work, between family and company: what are the issues for Southern countries? 4-6 October 2023 Marseille, France
Source: Med Trop Sante Int. 2024 Jun 7;4(2):mtsi.v4i2.2024.526. [Article in French] doi: 10.48327/mtsi.v4i2.2024.526 (PMC11292436; doi:10.48327/mtsi.v4i2.2024.526)
Supplement: Related texts

# Research article: La santé au travail à la lumière de l'histoire : construction sociale, enjeux politiques et fabrique de l'invisibilité à l'échelle globale

**Published:** 2024-06-07

**Authors:** Judith RAINHORN

**Affiliations:** Université Paris 1 Panthéon-Sorbonne, Chaire Recherche Santé-SHS

L’histoire de la santé au travail depuis le 19^e^ siècle éclaire l’invisibilité massive de ce fait social dans nos sociétés contemporaines. Dans de nombreux pays industrialisés, le processus de reconnaissance médico-légale des maladies professionnelles a donné lieu à un arsenal législatif, construit au cours des 19^e^ et 20^e^ siècles, qui s’avère aujourd’hui encore incapable de réparer de manière satisfaisante les maux du travail. La conférence éclairera la construction socio-politique de la reconnaissance des accidents du travail et des maladies professionnelles à partir de plusieurs cas européens, en montrant comment ce droit du travail s’est élaboré par àcoups, sous l’impulsion d’acteurs divers, et a contribué à la persistance d’une invisibilité massive. Elle mettra également en lumière les conditions de la construction des inégalités spatiales de santé à l’échelle globale depuis la création de l’OIT. Un coup d’œil dans le rétroviseur pour contribuer à comprendre les défis contemporains.

# Research article: Santé au travail dans le monde : de la CIST, à l'OMS/OIT aux archives des maladies professionnelles et de l'environnement

**Published:** 2024-06-07

**Authors:** Alexis D’ESCATHA

**Affiliations:** Université d’Angers, INSERM

La santé au travail dans le monde est un sujet porté depuis plus d’un siècle par la Commission internationale de santé au travail (CIST, ICOH en anglais) qui fédère les chercheurs comme les praticiens de nombreux pays et permet des échanges réguliers entre ces derniers (https://www.icohweb.org/site/homepage.asp). Certains de ses membres ont participé à une expertise conduite sous l’égide de l’OMS/OIT et qui a permis de fournir des résultats sur les principales maladies liées au travail dans le monde et prenant en compte des différences d’exposition dans les différents pays (https://www.who.int/publications/i/item/9789240034945) et qui vient d’être mis à jour : https://pubmed.ncbi.nlm.nih.gov/37265682.

Dans le monde de la santé au travail francophone, j’ai la chance de diriger les archives des maladies professionnelles et de l’environnement (Elsevier) et j’y ai développé une rubrique spécifique à la santé au travail hors de France. De nombreux articles permettent notamment d’insister sur la différence d’exposition et les moyens de prévention utilisés, comme en témoigne le récent numéro spécial qui y est consacré : https://www.sciencedirect.com/journal/archives-des-maladies-pro-fessionnelles-et-de-lenvironnement/special-issue/10X9QN2MJ6W.

# Research article: Malaria Control Programme (MCP) Sonamet, Lobito, Angola : du contrôle antipaludique dans l'entreprise au partenariat interuniversitaire 20 ans après.

**Published:** 2024-06-07

**Authors:** Patrick BESNARD

**Affiliations:** Sylvie MANGUIN IRD

En 1998, l’ETPM (Entreprise de travaux pétroliers maritime), filiale de GTM Entrepose, s’associe à l’entreprise angolaise d’État SONANGOL pour créer SONAMET, un chantier de construction parapétrolière situé dans la baie de Lobito, province de Benguela, à 500 km au sud de Luanda. En 2002, cette entreprise emploie sur ce chantier plus de 500 travailleurs dont le soutien médical est assuré par une petite équipe locale animée par un médecin français. Parmi les travailleurs, et dans la population locale, la pathologie dominante est le paludisme, à tel point que la maîtrise de sa transmission et de l’absentéisme massif associé devient une injonction prioritaire adressée par la direction au service médical.

Des actions empiriques sont amorcées par le service HSE, mais sans rien changer à la situation. Début 2003, le principe de l’organisation en vue de maîtriser cette situation est reformulé par le médecin, et présenté à la direction.

Il s’agit alors : 1) d’assurer un diagnostic fiable. 2) de réaliser des études entomologiques et de situer les lieux de transmission. 3) de créer une collaboration avec la santé publique, la situation endémique ne pouvant être modifiée localement dans l’enceinte du chantier. La direction de Sonamet valide le plan et demande au médecin de construire cette réponse.

Un premier effort vise à former des analystes capables d’assurer un diagnostic parasitologique de paludisme de qualité, puis de réaliser des explorations entomologiques. Des ingénieurs de recherches de l’OCEAC, avec le renfort d’un entomologiste médical de l’IRD, initient alors des formations, puis des études auprès des travailleurs.

En 2005, informé des premières actions développées et des premiers résultats transmis à la Santé publique, le directeur du PNCM (Plano

Nacional Contra Malaria) d’Angola, crée une relation de support avec l’équipe médicale de Sonamet et lui demande de s’investir dans une région fortement endémique, Balombo à 150 km de Lobito.

Depuis le début du processus, de nombreuses actions et études, impliquant notamment des experts de l’IRD, seront rapportées dans une vingtaine d’articles durant 20 ans. Observations et résultats seront partagés avec l’OMS. Ce système de contrôle antipaludique, « monitorage » d’une équipe implantée sur le terrain supervisée par le médecin et des experts scientifiques internationaux présents par intermittence, fonctionnera de 2004 jusqu’à aujourd’hui. Il est original, sinon unique, et représente un modèle performant également par sa sobriété économique.

# Research article: Dernières évolutions de la santé au travail en France : quelle substantifique moëlle pour les pays du sud ?

**Published:** 2024-06-07

**Authors:** Fabrice MICHIELS

**Affiliations:** Service Prévention Santé Travail Corrèze-Dordogne

Le dispositif dédié à la santé au travail français est considéré comme l’un des plus ambitieux et abouti. Objet de réformes itératives, ses principes fondamentaux demeurent d’une part l’entreprise, avec un employeur dépositaire d’une obligation de résultats, des salariés et leurs représentants et, d’autre part, des intervenants extérieurs les assistant dans leur démarche de prévention des risques professionnels, en particulier les services de prévention et santé au travail (SPST), dont la mission principale est d’éviter toute altération de la santé du fait du travail.

L’action des SPST s’inscrit dans quatre grandes missions que la dernière réforme du dispositif, issue d’une loi du 02/08/21, clarifie et vise à harmoniser, au travers d’une « offre socle » nationale, tout en les élargissant et en décloisonnant la santé au travail.

La prévention primaire des risques professionnels est la principale mission. Elle impose de repérer les dangers présents dans les entreprises, en s’appuyant sur une large veille scientifique et technologique, de conseiller sur les mesures de prévention à mettre en œuvre, ceci étant retranscrit dans la fiche d’entreprise, mais aussi d’aider les employeurs à évaluer les risques et à élaborer un plan d’action, objet du Document unique d’évaluation des risques. Les SPST doivent pour cela effectuer une action de prévention primaire au minimum quadriennale pour chaque entreprise.

La 2^e^ mission s’articule autour du suivi individuel de l’état de santé des « travailleurs », incluant désormais dirigeants non-salariés et travailleurs indépendants. Visant théoriquement avant tout à informer les travailleurs sur les risques qu’ils encourent et les moyens de les prévenir, ce suivi reste centré sur l’aptitude. Le cadre imposé découle bien davantage de besoins juridiques de conformité pour les entreprises, que d’un raisonnement médical et scientifique. La dernière réforme met l’accent sur deux axes louables : améliorer le dépistage et le suivi des effets des expositions professionnelles, et la prévention de la désinsertion professionnelle.

Le maintien dans l’emploi constitue d’ailleurs le 3^e^ axe fort de l’offre socle des SPST. Loin d’être une mission nouvelle, les exigences en termes d’organisation interne et de partenariats externes sont renforcées elles aussi.

L’expérience française est riche d’enseignements pour qui souhaiterait développer un système similaire. La réglementation, tout en fixant les objectifs généraux du dispositif, devrait laisser aux spécialistes que sont les médecins du travail le choix des modalités concrètes de suivi des travailleurs, sur la base de *ïevidence based medicine,* comme toute autre spécialité médicale.

# Research article: Surveillance de la santé des militaires français exerçant outre-mer

**Published:** 2024-06-07

**Authors:** Jean-Marie LOREAU

**Affiliations:** Assistant de santé publique, CESPA (Centre d’épidémiologie et de santé publique des armées), Marseille.

La protection de la santé de la force (PROSANFOR) est une collaboration entre de nombreux partenaires qui vise à maintenir le potentiel humain, individuel et collectif des personnels militaires et promouvoir ou conserver leur bien-être physique et mental. Le Service de santé des armées y contribue, avant, pendant et après l’engagement, en tout temps et partout où des militaires français sont déployés : plus de 320 antennes médicales en France métropolitaine et ultramarine, à l’étranger et sur les bâtiments de la Marine permettent des actions de prévention, de soutien, de prise en charge médicale et chirurgicales. Où qu’ils soient, les militaires sont exposés à des risques inhérents à leur métier, mais ils sont aussi exposés, comme les civils, à de multiples risques infectieux ou environnementaux.

La surveillance épidémiologique dans les armées est un pilier de la PROSANFOR. Elle repose sur la déclaration immédiate de plus de 60 évènements de santé : les maladies à déclaration obligatoire du code de la santé publique français, d’autres maladies infectieuses pouvant avoir un impact particulier dû à la vie en collectivité ou pouvant être le témoin d’une émergence, et des pathologies d’intérêt spécifique au milieu militaire. Le service de surveillance épidémiologique et investigation basé à Marseille au sein du Centre d’épidémiologie et de santé publique des armées (CESPA) coordonne et s’assure du bon fonctionnement de la chaîne de déclaration via un système d’information dédié, OSEA, déployé dans toutes les antennes médicales. Il centralise, vérifie, et interprète, en temps réel, toutes les déclarations, déclenche, si nécessaire, des investigations plus poussées et soutient la mise en œuvre des actions de riposte aux épidémies ou risque épidémique. Il fournit aussi des données épidémiologiques indispensables au pilotage des crises sanitaires ou des plans de santé publique, et à l’identification des priorités de santé et de recherche.

Le système mis en place permet de surveiller les émergences en milieu militaire et d’informer les autorités sanitaires locales de l’apparition d’événements pouvant avoir un impact sur les populations civiles locales, comme ce fut le cas lors de l’épidémie de chikungunya à Djibouti fin 2019. L’intervention pluridisciplinaire (infectiologue, biologiste, santé publique, entomologiste) a permis de diminuer l’impact de l’épidémie au sein des forces françaises stationnées à Djibouti et de la communauté de défense française.

En outre-mer, l’événement le plus souvent déclaré est les diarrhées aigües, pouvant avoir un impact important sur la capacité opérationnelle des forces, montrant l’importance de l’hygiène en prévention primaire.

# Research article: Conditions de travail des militaires français exerçant outre-mer : impact et prévention

**Published:** 2024-06-07

**Authors:** Aurélie MAYET

**Affiliations:** Professeur agrégé du Val-de-Grâce, CESPA (Centre d’épidémiologie et de santé publique des armées), Marseille.

Outre-mer ou en opérations extérieures (OPEX), les militaires sont confrontés non seulement aux conséquences des actions de combat, mais aussi à de nombreux événements de santé. Selon l’enquête nouvelle génération, conduite en 2019 auprès de 4 266 militaires, 22 % de ceux ayant été au moins une fois en mission extérieure dans les cinq dernières années déclaraient avoir eu un problème de santé au cours de la mission et 8 % en avoir eu plusieurs. Les principaux problèmes de santé rencontrés étaient les problèmes infectieux (diarrhées, fièvre ou paludisme), les problèmes dermatologiques et la traumatologie faisant suite à des accidents. Les répondants identifiaient cinq thèmes de santé prioritaires en matière de prévention en OPEX : les maladies à transmission vectorielle, le stress, l’hygiène, les troubles psychiques incluant les états de stress post-traumatique et la dépression, et les conditions climatiques. D’autres travaux récents portant sur les évacuations médicales stratégiques retrouvaient, comme principales causes d’évacuation, les pathologies médicales (notamment des crises de coliques néphrétiques), les traumatismes (blessures de guerre, accidents de la voie publique…) et les affections psychiatriques à type de troubles de l’adaptation et d’états de stress aigu.

En OPEX, les militaires sont en effet confrontés à de nombreuses expositions physiques (chaleur, humidité, soleil.), psychiques (stress, ennui, isolement familial.), biologiques (maladies infectieuses) et chimiques (risque industriel, exposition aux pesticides.). Il apparaît aussi qu’une importante partie des événements de santé auxquels sont confrontés les militaires déployés est étroitement liée aux comportements individuels : usage de substances psychoactives, prise de risque routière, mauvaise observance aux mesures de lutte antivectorielle. La prévention de la plupart de ces événements repose sur l’application des principes d’hygiène en opérations (hygiène de l’eau, de la nourriture, des matières usées, du bien-être mental.), souvent difficile en contexte opérationnel, et la mise en place d’actions de promotion de la santé. Enfin, si l’impact à court-terme de certaines expositions opérationnelles sur la santé est dans l’ensemble quantifiable (maladies infectieuses, coliques néphrétiques sur déshydratation, troubles psychiques de l’adaptation.), les conséquences à long terme de ces expositions restent méconnues et leur étude constitue un défi méthodologique : suivi prospectif à long terme de militaires, même après la fin de la carrière, mesure de nombreuses expositions professionnelles et personnelles. La mise en place d’une cohorte généraliste de militaires dans les prochaines années par le Service de santé des armées pourrait apporter de premiers éléments de réponse.

# Research article: Santé au travail des porteurs népalais employés par l'industrie touristique

**Published:** 2024-06-07

**Authors:** Dominique JEAN

**Affiliations:** Pédiatre, infectiologue, himalayiste

Les premières expéditions vers les sommets himalayens ont naturellement employé comme porteurs des Sherpas. D’origine tibétaine, vivant dans la région de l’Everest, ils étaient parfaitement adaptés à l’altitude, habitués à porter des charges lourdes dans leurs activités quotidiennes et ce travail qui leur paraissait facile leur permettait de toucher un salaire. Les touristes sont arrivés plus tard et la région de l’Everest a été ouverte au trekking en 1970. Le mal des montagnes était très mal connu à l’époque et il y avait 5 à 10 décès par an pour un nombre de randonneurs inférieur à 1 000. Un poste de secours rudimentaire s’est installé à 4 250 m et l’*Himalayan Rescue Association* a été créée en 1973. Les Sherpas éduqués s’étant enrichis, les porteurs actuels sont issus de populations pauvres de basse altitude. Recrutés au départ d’un trek, ils ne sont pas adaptés à l’altitude, et doivent s’acclimater comme les touristes. Ne voulant pas risquer de perdre leur emploi, ils ne disent rien quand ils sont malades et on peut les retrouver morts d’un œdème pulmonaire au bord du chemin. Ils peuvent également souffrir d’ophtalmie des neiges ou de gelures si les touristes ne leur fournissent pas les équipements adaptés. En 1997, suite au décès d’un porteur par œdème d’altitude, un médecin britannique a créé l’IPPG : International Porter Protection Group. En 2002, l’association a ouvert un poste médical à 4 450 m dans la vallée de Gokyo avec l’accord du Parc national de l’Everest et du gouvernement local. Un 2^e^ poste a été ouvert à 4 800 m en 2011. Les soins étaient gratuits pour les porteurs et payants pour les touristes, ce qui permettait un équilibre budgétaire, et tout a bien fonctionné pendant 17 ans. Mais en 2018 un système de corruption généralisée a été révélé, impliquant des agences touristiques, des hôteliers, des compagnies d’hélicoptères et des cliniques, tous ayant pour but d’évacuer le maximum de touristes vers Katmandu en leur faisant croire qu’ils allaient mourir du mal d’altitude, réalisant une vaste arnaque aux assurances. En 2019, une clinique privée a ouvert à Gokyo, et le jeune médecin employé évacuait 50 % des touristes vers la clinique correspondante à Katmandu avec la compagnie d’hélicoptères associée, alors qu’habituellement seulement 5 % des touristes étaient évacués. Et les soins aux porteurs étaient devenus payants ! Les deux postes bénévoles de l’IPPG ont alors eu l’interdiction d’ouvrir au printemps 2020, et la pandémie Covid a empêché tout recours.

# Research article: Vulnérabilité sanitaire et sociale des travailleurs du déchet dans la Vallée de Katmandu : une recherche-action de Médecins du monde

**Published:** 2024-06-07

**Authors:** Astrid FOSSIER-HECKMANN

**Affiliations:** Santé publique. Médecins du Monde : groupe Santé Environnement

Une étude transversale sur la santé des travailleurs de déchets dans la vallée de Katmandu au Népal a été réalisée par l’Université de Sheffield, Médecins du monde et l’association népalaise Phase en 2017 sur un échantillon de 1 278 travailleurs.

Cette étude démontre que les travailleurs de déchets informels ont un risque accru d’accidents sur leur lieu de travail et qu’ils bénéficient de peu de mécanismes de protection et de prévention, tels que la vaccination ou le port d’équipements de protection.

En outre, la vulnérabilité de cette population est accrue par leur situation socio-économique marginale, leur manque d’accès à l’information et aux soins et les discriminations qu’ils subissent dans un système social très hiérarchisé (castes et ethnicité).

Depuis 2016, Médecins du monde travaille auprès de cette population dans une approche de renforcement communautaire et de promotion de la santé, afin de les accompagner dans leur accès aux soins, aux droits, et à une reconnaissance par les autorités.

# Research article: Problèmes de santé au travail chez les tanneurs de Fès

**Published:** 2024-06-07

**Authors:** Sanae ACHOUR

**Affiliations:** Professeur de pharmacologie-toxicologie, Université Sidi Mohamed Ben Abdellah, Fez

Le processus de tannage du cuir est bien connu pour l’utilisation intensive de produits chimiques et de matières biologiques qui posent de sérieux problèmes pour la santé humaine, l’environnement et la communauté entière. L’objectif principal de ce travail de thèse était d’étudier l’impact des conditions de travail sur la santé des tanneurs exerçant dans les tanneries traditionnelles de Fès, Maroc. Ce travail a permis également d’évaluer le taux de chrome sanguin chez les tanneurs par comparaison avec les concentrations observées dans la population générale de Fès, non exposée professionnellement.

Cette étude transversale a été réalisée entre octobre 2017 et janvier 2019, dans les tanneries traditionnelles de Fès. Au total, 400 tanneurs artisans ont donné leur accord de participation à l’étude des postes et des conditions de travail, soit un taux de participation de 50 %. Parmi ces 400 personnes travaillant dans les trois tanneries interrogées, 220 tanneurs ont bénéficié des examens médicaux, des bilans sanguins et urinaires et ont été comparés à 220 témoins. Le bilan sanguin comprenait des analyses hématologiques, biochimiques et toxicologiques (chrome sanguin). Le bilan urinaire comprenait un examen cytobactériologique des urines. Le chrome sanguin total a été mesuré par ICPMS et l’analyse statistique a été effectuée à l’aide du logiciel SPSS V20.

L’enquête sur le lieu du travail a révélé des conditions de travail et d’hygiène défavorables associées à l’absence d’équipement de protection individuelle (EPI). Les postures de travail, l’exposition directe aux produits chimiques et aux risques biologiques seraient à l’origine de plusieurs maladies professionnelles. Les troubles musculo-squelettiques (TMS) 50,9 %, génito-urinaires (39,5 %), dermatologiques (30,4 %), oculaires (24,5 %) et pulmonaires (16,8 %) étaient fréquemment rencontrés chez les tanneurs artisans.

La fréquence des infections urinaires était légèrement plus élevée (3,4 %) chez les tanneurs par rapport aux témoins (0 %). L’analyse multivariée a montré que le travail en milieu humide était le principal facteur qui peut augmenter le risque de développer les TMS et les atteintes dermatologiques. L’exposition aux produits chimiques et aux risques biologiques peuvent aussi augmenter le risque de développer les atteintes oculaires et génito-urinaires respectivement. Sur le plan biologique, certaines variations ont été notées dans les niveaux des globules blancs (GB), d’aspartate aminotransférase (AST), d’alanine aminotransférase (ALT), de créatinine et de glucose. Cependant, ces variations étaient très faibles et probablement sans signification clinique. Le pourcentage de détection du chrome dans le sang était plus faible chez les tanneurs par rapport aux témoins (4,1 % vs. 24,5 %); ce qui confirme l’absence d’exposition au chrome dans les tanneries traditionnelles.

En conclusion, cette étude a révélé une prévalence élevée de certaines pathologies chez les tanneurs artisans de Fès. Par ailleurs, aucune preuve d’effets hématologiques et biochimiques indésirables liés à l’imprégnation par le chrome n’a été observée.

# Research article: État de santé et prise en charge des vendeurs ambulants à Bangui (République centrafricaine)

**Published:** 2024-06-07

**Authors:** Hermione Dahlia MOSSORO-KPINDE

**Affiliations:** Médecin du travail, Directrice de la médecine du travail au ministère du Travail, de l’Emploi, de la Protection sociale et de la Formation professionnelle, Bangui, RCA

## Contexte et objectif

La vente ambulante est une activité économique en pleine expansion dans les pays à faibles revenus dont l’importance et l’état de santé des acteurs ne sont pas connus. La RCA est en train d’élaborer le plan national de protection sociale. L’objectif de l’étude était d’évaluer l’état de santé des vendeurs ambulants à Bangui ainsi que leur prise en charge.

## Matériel et méthodes

Une étude transversale descriptive et analytique incluant les vendeurs ambulants de Bangui a été réalisée du 1er septembre à 31 octobre 2021. Les données sociodémographiques, économiques et sanitaires ont été collectées par interview grâce à un questionnaire d’auto-évaluation de l’état de santé.

## Résultats

Au total, 384 vendeurs ambulants ont été inclus, âgés en moyenne de 27 ans, avec une prédominance masculine : le sexratio était de 3,0. Ils étaient essentiellement célibataires (98,4 %), de niveau d’instruction secondaire (55,2 %). 64 % d‘entre eux avaient une famille à charge. La vente ambulante (ambulantage) était l’unique source de revenu dans 86,5 % des cas. Le bénéfice journalier était compris entre 100 et 3 000 FCFA chez 44 % de notre échantillon. Les 2/3 travaillaient 7 à 12 heures par jour (64,8 %) et parcouraient plus de 10 km par jour à pied en portant leur marchandise (69,3). Les principaux problèmes de santé signalés étaient les troubles musculosquelettiques (95,8 %), les troubles de comportement (55,7 %), les maladies infectieuses (53,4 %) et les troubles respiratoires (26,0 %); 12,5 % avaient déjà fait au moins une fois le dépistage pour le coronavirus, dont 23,9 % (22) étaient positifs. La vaccination contre le Covid 19 avait été réalisée par 2,6 % des enquêtés. Seulement 27,6 % (106) allaient en consultation médicale, en cas de maladie et 1,8 % pour un bilan annuel. Tous évoquaient le manque d’argent comme obstacle et 88 % prenaient des médicaments traditionnels. La prise de médicaments modernes se faisait en automédication par 80 % des enquêtés. Parmi ces médicaments les anti-inflammatoires étaient les plus consommés 21,8 % (84) suivi des antibiotiques 13,5 % (52). Aucun n’était affilié à un système de protection sociale.

## Conclusion

Les résultats de cette étude montrent que les vendeurs ambulants ont un poids économique certain, mais leurs activités impactent souvent négativement leur état de santé. Leur revenu ne leur permet pas souvent une prise en charge de leurs pathologies par des professionnels de la santé. Ces résultats suggèrent de les organiser et de leur assurer une protection sociale adéquate.

# Research article: Épidémiologie et prise en charge du burnout dans les entreprises au Cameroun

**Published:** 2024-06-07

**Authors:** Léon Jules OWONA MANGA

**Affiliations:** Maître de conférences agrégé, médecin du travail, Faculté de médecine et des sciences pharmaceutiques de Douala, Cameroun

Le burnout est le syndrome d’épuisement physique et émotionnel qui affecte principalement les travailleurs des services d’aide à la personne et des sociétés de services. Il est consécutif aux nombreuses mutations qui ont affecté l’organisation et les conditions de travail au cours des dernières décennies. Réel problème de santé publique dans les pays développés, sa fréquence est en augmentation dans le monde, et certains auteurs considèrent le burnout comme le mal des sociétés modernes et un défi pour la recherche et la pratique.

Cette communication a pour but de partager notre expérience de la connaissance et de la prise en charge du burnout en contexte africain.

Au plan épidémiologique, un intérêt de la communauté scientifique pour le burnout s’est traduit par d’importants travaux de recherche qui ont abouti à la rédaction de milliers d’ouvrages, de chapitres de livres, de thèses et d’articles originaux publiés.

Dans notre contexte, les mères de famille, les célibataires âgées de 30-45 ans et les employés administratifs, les cadres et les agents de maîtrise en sont les principales victimes. Au plan socioprofessionnel, la surcharge de travail, les mauvaises conditions de travail, la conciliation difficile entre la vie familiale et la vie professionnelle, ainsi que les humiliations constituent des facteurs de risque du burnout.

Les sociétés de services (électricité, téléphonie), les grandes entreprises, l’enseignement supérieur, les ONG internationales sont les principaux pourvoyeurs de cas connus de burnout.

La prise en charge du burnout sera multidisciplinaire en raison de l’origine généralement mixte, sociale et professionnelle. Elle va requérir l’intervention d’une diversité d’acteurs internes et externes à l’entreprise. Elle consistera principalement au retrait de l’activité, au repos médical (durée plus ou moins longue), au suivi médical et psychologique, à des changements positifs dans le milieu de travail et en l’assistance psycho-affective pour le conjoint, la famille, les enfants, la psychothérapie.

Une ligne verte (1510) : Centre d’assistance psychologique nationale a été ouverte par le ministère de la Santé publique.

# Research article: Prise en charge des employés de grandes entreprises africaines ayant contracté le Covid 19 durant leur activité professionnelle

**Published:** 2024-06-07

**Authors:** Erik MAVILLE

**Affiliations:** Economiste, directeur général Santé En Entreprise (SEE)

La crise sanitaire et d’autres crises mondiales ont effacé en 3 ans une partie des progrès enregistrés au cours des 15 dernières années dans le combat contre les grandes endémies (VIH, tuberculose, paludisme), sans compter de nouveaux défis à relever en matière de prévention et de dépistage précoce des maladies non transmissibles (diabète, maladies cardiovasculaires, cancers…) Les entreprises internationales opérant en Afrique, en tant qu’acteurs économiques et « employeurs responsables » sont appelées à jouer un rôle plus actif dans le domaine de la santé mondiale, Ce faisant, elles cherchent à intégrer la santé dans leur « feuille de route RSE » dans le cadre d’une approche articulant santé au travail, santé publique et santé environnementale.

Comment l’implication des entreprises a-telle évolué dans le domaine de la santé depuis les 20 dernières années en Afrique ?

La nécessité d’une approche « One Health » intégrée est désormais reconnue. Mais où en sommes-nous réellement dans le passage à l’action ?

Comment la médecine du travail s’inscritelle dans le cadre de cette nouvelle gouvernance de la santé en entreprise (SEE) ?

Quels sont les secteurs économiques et les entreprises les plus dynamiques en matière de promotion de la santé ? Quelles sont concrètement les stratégies utilisées pour passer à l’action ? Quels sont les moyens mobilisés pour opérationnaliser les feuilles de route « RSE », mesurer l’impact des actions, évaluer leur contribution à la réalisation des ODD et rendre compte aux parties prenantes ? Comment éviter le risque de « Social Washing » ?

# Research article: Prise en charge des pathologies des travailleuses du sexe au Burkina Faso : le cas de la Cohorte Yerelon « Étude ANRS 1222 »

**Published:** 2024-06-07

**Authors:** Isidore TRAORE

**Affiliations:** Médecin, épidémiologiste, Institut Supérieur des Sciences de la Santé (Université Nazi Boni) et au Centre MURAZ (Institut National de Santé Publique), Bobo-Dioulasso, Burkina Faso

Au Burkina Faso, la prévalence du VIH est 5 à 10 fois plus élevée chez les travailleuses du sexe (TS) qu’en population générale. Les interventions ciblant ce groupe vulnérable, sont donc une composante importante de la réponse de santé publique aux infections sexuellement transmissibles (IST) et au VIH. De 1998 à 2011, grâce à un financement de l’Agence française de recherches sur le sida et les hépatites virales (ANRS) le programme de recherche (Étude ANRS1222) « Yerelon » (qui signifie « se connaître soi-même » en langue bambara) a été mis en œuvre chez les TS dans la ville de Bobo-Dioulasso (capitale économique du Burkina Faso). Il comprenait une intervention basée sur une approche par les pairs, une clinique spécifique conviviale prodiguant des soins de base gratuits, une organisation des TS en association, le soutien social et des études internes et externes de satisfaction des participantes. Un schéma d’étude de cohorte ouverte a été adopté pour la mise en œuvre de cette intervention et l’évaluation de son efficacité. L’intervention a été testée et validée dans une autre ville (Ouagadougou).

Durant la phase formative (de 1998 à 2002) l’incidence du VIH était de 3 pour 100 personnes-années. Durant l’intervention (Yerelon2 à Yerelon4), sur les 917 participantes incluses dans les cohortes, 38 % (352/917) étaient séropositives au VIH à l’inclusion. Le taux de rétention était de 89 %. Ce taux était supérieur à 95 % après 2006. Le principal motif de perdu de vue était le voyage hors de la ville de Bobo-Dioulasso.

Le programme « Yerelon, » a permis de décrire la typologie des femmes impliquées dans le commerce du sexe à Bobo-Dioulasso, d’estimer la prévalence et l’incidence d’autres IST, d’évaluer l’impact de l’intervention sur la transmission du VIH et l’efficacité du traitement antirétroviral (ARV) chez les TS et l’impact du traitement anti herpétique sur la réduction de la charge virale VIH chez les TS co-infectées par VIH et HSV-2.

Des études sur les infections par le HPV et le cancer du col de l’utérus, des évaluations économiques et socio-anthropologiques ainsi que la modélisation de l’impact du paquet d’interventions sur la réduction de l’incidence du VIH dans la population générale ont aussi été réalisées.

En conclusion, la cohorte Yerelon (ANRS 1222) a permis d’élaborer et de tester une stratégie efficace de prévention et de prise en charge du VIH chez les femmes vulnérables.

# Research article: Pathologies liées à l'exploitation aurifère et accès aux soins des travailleurs clandestins en Guyane

**Published:** 2024-06-07

**Authors:** Maylis DOUINE

**Affiliations:** Médecin, chercheuse en santé publique, Centre hospitalier de Cayenne, Guyane

La santé des individus et des communautés résulte de la combinaison de différents déterminants tels que l’environnement politique, social et économique, l’environnement physique, les caractéristiques et comportements individuels et l’accès aux soins. En Guyane, une population particulièrement vulnérable vit à l’écart du système de soins : celle des personnes travaillant sur les mines d’or illégales au cœur de la forêt tropicale. Ces personnes, plus de 10 000, sont majoritairement originaires du Brésil et travaillent clandestinement sur plusieurs centaines de sites d’orpaillage illégaux au cœur de la forêt tropical guyanaise.

Leur mode de vie en forêt amazonienne entraîne de nombreux problèmes de santé. La présence de vecteurs (anophèles, *Aedes,* phlébotomes) les expose au paludisme, à la leishmaniose, aux arboviroses. Le contact étroit avec la biodiversité amazonienne favorise la circulation des zoonoses comme la leptospirose ou la brucellose, et le passage de barrière d’espèce de pathogènes potentiellement émergents comme des anaplasmoses. L’absence d’accès à l’eau potable et aux latrines entraîne des maladies du péril fécal. L’utilisation du mercure pour amalgamer les particules d’or expose ces travailleurs à des pathologies respiratoires et les populations, notamment amérindiennes, vivant en amont à des troubles du développement des enfants. L’éloignement du système de soin et leur statut illégal sur le territoire entraîne des retards de prise en charge, des pertes de suivi et un recours fréquent à l’automédication avec un risque d’émergence de résistances.

Les orpailleurs illégaux cumulent donc de nombreux risques sanitaires du fait de leur mode de vie et de leur activité. Cette activité illégale pose des problèmes écologiques, économiques et sociétaux. Cependant, pour des raisons éthiques ainsi que de santé publique, des actions de promotion de la santé devraient être discutées à différents niveaux : individuel, environnemental, systémique et global.

# Research article: Santé en entreprise : enjeux socio-économiques dans les pays du Sud

**Published:** 2024-06-07

**Authors:** Ubald ASSOGBA-GBINDOUN

**Affiliations:** Néphrologue, praticien hospitalier, Conseil des Béninois de France et Forum des Organisations de Solidarité Internationale issues des Migrations (FORIM)

Le développement des entreprises de façon structurée reste la clé d’une possibilité de mise en place des éléments d’une amélioration socio-économique des travailleurs des pays du Sud. La mise en place de l’assurance qualité au sein des entreprises ouvre la voie d’un développement durable en matière santé surtout quand on y met les indicateurs de suivi et qualité de la santé du personnel. L’assurance maladie obligatoire demandée par l’Etat et la mutuelle instaurée et négociée au sein d’une entreprise est un acte socialement fort et économiquement rentable pour l’entreprise et pour le pays. Cet acte fort inclut le travailleur et sa famille sur le plan de la surveillance santé, car en plus d’être un acteur de développement économique il associe le facteur du progrès social.

# Research article: Incidence des morsures de serpent dans les communautés rurales de savane de Paoua et de forêt de Mbaïki en République centrafricaine

**Published:** 2024-06-07

**Authors:** Romaric Ghislain ZARAMBAUD BOHY-NGOMBET

**Affiliations:** École doctorale des sciences de la santé humaine et vétérinaire de l’Université de Bangui (RCA)

## Introduction

L’objectif de cette étude est de décrire et de comparer les aspects épidémiologiques des morsures de serpent parmi les populations vivant dans le milieu rural de la zone de savane de Paoua et de la zone de forêt de Mbaïki en République centrafricaine (RCA).

## Matériels et méthodes

Il s’agit d’une étude prospective à base communautaire dans deux districts sanitaires en RCA, de décembre 2019 à janvier 2021. Tout cas de morsure de serpent rapporté par les enquêteurs communautaires a fait l’objet d’un questionnaire afin de déterminer l’incidence, les circonstances de survenue, la gravité de la morsure et l’itinéraire thérapeutique.

## Résultats

Au total, 412 cas de morsures de serpent ont été recensés au cours de l’étude dont 198 cas dans la zone forestière et 214 dans la zone de savane. La létalité était de 5 % dans la zone de forêt et de 1 % dans la zone de savane.

Le taux d’incidence des morsures de serpent était nettement plus élevé chez les enfants de savane en comparaison avec ceux de la forêt (98/100 000 vs 25,1/100 000; p < 0,00001), tandis que ce taux d’incidence était nettement plus bas à partir de 45 ans dans la zone de savane de Paoua comparativement à la zone de forêt de Mbaïki (167/100 000 vs 200/100 000; p = 0,02). La létalité des enfants et des adultes jusqu’à 44 ans apparaissait nettement plus élevée en zone de forêt (sept décès *vs* un décès).

Ces morsures survenaient beaucoup plus lors des activités agricoles dans la zone de savane que dans la zone de forêt (51 % *vs* 26 %, p < 0,0001).

La symptomatologie était dominée par l’œdème et le saignement au point de morsure dans les deux zones d’études, compatibles avec un syndrome vipérin.

## Conclusion

Avec un taux d’incidence supérieur à 160 cas pour 100 000 habitants dans la population active de 15-44 ans des communautés rurales de la zone de forêt et de savane, les morsures de serpent restent un problème de santé publique en RCA. Une étude sur les espèces et la toxicité de leurs venins en RCA est recommandée. Par ailleurs, il est urgent de rendre disponible les sérums antivenimeux dans les formations sanitaires périphériques afin de réduire la mortalité liée à ces envenimations.

# Research article: Identification des serpents responsables des envenimations au Maroc. Données du Centre antipoison et de pharmacovigilance du Maroc de 2008 à 2018

**Published:** 2024-06-07

**Authors:** Fouad CHAFIQ, Mohamed FEKHAOUI, Abderrahman MATAAM, Soulaymani BENCHEIKH

**Affiliations:** Centre antipoison et de pharmacovigilance du Maroc - Rue Lamfadel Cherkaoui, Rabat-Instituts, B.P 6671, Rabat, Maroc

## Introduction

Au Maroc, sur 28 espèces de serpents rencontrées, 8 espèces présentent un réel danger pour la population. La létalité est estimée à 7,2 %. Depuis 2008, le CAPM a mis en place une stratégie de lutte contre les envenimations. Parmi les axes stratégiques développés est la reconnaissance des serpents venimeux responsables des envenimations.

## Objectif

Déterminer à partir d’une étude rétrospective de l’activité de l’identification de spécimens de serpents par le CAPM, les serpents responsables des envenimations au Maroc.

## Matériel et méthodes

C’est une étude rétrospective à visée descriptive sur une période de 11 ans allant de 2008 à 2018. Elle a concerné toutes les demandes d’identification de serpent venimeux au CAPM après morsure.

## Résultats

Durant notre étude, 143 identifications de spécimen de serpent responsables de morsures ont été recensées sur un total de 2703 de morsures notifiées ce qui représente 5,3 %. Deux familles de serpents sont identifiées : les vipéridés représentaient 93.7 % (n = 134) et les élapidés représentés par une seule espèce *Naje haje legionis* ou cobra ont été identifiés dans 6,3 % (n = 9). L’envoi d’images de serpents agresseurs par téléphone portable au CAPM représentait 72.8 % des modalités d’identification. Parmi les vipères identifiées, *Daboia mauritanica* représentait 63,6 % (n = 91) et sa répartition géographique englobe une grande partie du Maroc. *Cerastes cerastes* représentait 17,5 *%* (n = 25) et a été identifiée dans le sud et sudest du Maroc. *Bitis arietans* représentait 9,8 % (n = 14) et a été identifiée dans les franges côtières du sud du Maroc. *Echis leucogaster* a été identifiée dans 1,4 % (n = 2). *Vipera latastei* et *Vipera montícola* ont été identifiées chacune dans un seul cas et ont été identifiées respectivement dans le nord et le moyen Atlas. L’âge moyen des patients était de 34,3 ans avec un sex-ratio = 1,58 en faveur du sexe masculin. Les morsures par vipéridés ont été responsables du syndrome vipérin avec un taux d’envenimation de 95,3 % et la thrombopénie a été observée dans 60 % (69/115). Les morsures par cobra étaient responsables de tableau neurologique dans 77,7 %. Les envenimations fatales ont été observées à la suite de morsures par *Daboia mauritanica* (8 décès*), Cerastes cerastes* (2 décès*) et Naja haje* (3 décès).

## Conclusion

L’instauration de l’activité d’identification ophidienne par le CAPM et la sensibilisation des professionnels de santé a permis une meilleure reconnaissance des serpents venimeux présentant un intérêt médical au Maroc qui sont *Daboia mauritanica, Cerastes cerastes, Bitis arietans et Naja haje.* Cette reconnaissance a permis aussi de cibler les actions de sensibilisation de la population et l’amélioration de la prise en charge des envenimés dans les zones à risque.

# Research article: Premier cas autochtone d'arbovirose due au virus Usutu en Nouvelle-Aquitaine : description et mesures de gestion

**Published:** 2024-06-07

**Authors:** Karim TARARBIT, Anne BERNADOU, Christophe MATRAS, Baptiste GROFF, Guillaume LACOUR, Gilda GRARD, Denis MALVY, Marie-Claire PATY, Laurent FILLEUL, Sylvie QUELET

**Affiliations:** ARS Nouvelle Aquitaine - Cellule de Veille Alerte et Gestion Sanitaire - Pôle Veille Sanitaire et Prévention du Risque Infectieux - Direction déléguée à la Santé Publique et aux Environnements; Direction de la Protection de la Santé et de l’Autonomie - 103 bis rue Belleville. CS 91704. 33063 Bordeaux, France

## Introduction

Une surveillance renforcée du chikungunya, de la dengue et du Zika est active entre mai et novembre dans tous les départements de métropole : tous les cas confirmés ou probables, importés ou autochtones doivent être signalés à l’Agence régionale de santé (ARS). Le 07/10/2022, un cas suspect autochtone de dengue a été signalé à l’ARS Nouvelle-Aquitaine. Les objectifs des investigations étaient de confirmer le caractère autochtone, déterminer le virus en cause et mettre en place les mesures adaptées.

## Matériels et méthodes

L’enquête épidémiologique a recueilli l’histoire clinique, recherché des expositions à risque et la présence d’autres cas suspects dans l’entourage. L’enquête entomologique a recherché la présence de vecteurs sur les lieux fréquentés en période d’exposition et de virémie. Le Centre national de référence des arbovirus (CNR) a réalisé la confirmation diagnostique à partir de prélèvements du 26/09 et du 11/10.

## Résultats

La patiente a présenté le 18/09 un syndrome pseudo-grippal et une éruption cutanée, sans signe de gravité. Elle n’avait pas séjourné hors de Nouvelle-Aquitaine pendant sa période d’exposition. Aucun cas suspect n’a été retrouvé dans son entourage. Le 17/10, le CNR a confirmé sur le 1^er^ prélèvement une infection récente à flavivirus, sans diagnostic d’espèce en l’absence de contribution de la recherche de matériel viral. Le contexte épidémiologique et le tableau clinique faisaient évoquer en priorité les diagnostics de dengue et de West Nile. Sans attendre le diagnostic virologique précis, des mesures de gestion ont été déployées : lutte anti-vectorielle ciblant les moustiques adultes et gîtes larvaires à *Aedes* et *Culex,* sensibilisation des médecins et laboratoires, information et sécurisation du dispositif de dons biologiques. Le 04/11, le CNR a confirmé, par séroneutralisation sur le 2^nd^ sérum, le diagnostic d’infection récente à virus Usutu. L’infection humaine à Usutu étant une impasse épidémiologique, aucune autre mesure de gestion n’était justifiée. Aucun autre cas humain n’a été identifié par la suite.

## Conclusion

Il s’agit du premier cas humain autochtone d’infection à Usutu identifié en Nouvelle-Aquitaine et le 2^e^ cas rapporté en France. Il survient après la détection des premiers cas aviaires d’infection à Usutu en Nouvelle-Aquitaine en septembre 2022. L’infection à Usutu est une arbovirose émergente : une extension de l’aire géographique de présence du virus au sein de l’avifaune sauvage et chez le vecteur est observée en Europe. Cet évènement confirme l’intérêt de poursuivre la surveillance épidémiologique des arboviroses d’une manière réactive et cela dans une approche One Health.

# Research article: Encéphalite aiguë à *Rickettsia conorii* : à propos d'une série de 15 cas

**Published:** 2024-06-07

**Authors:** Bouchaib HAYET, Achour AMRANE, Philippe PAROLA

**Affiliations:** CHU Tizi-ouzou. Algérie

La rickettsiose est une zoonose transmise à l’homme par des arthropodes. Elle est généralement bénigne. Cependant, des formes neurologiques graves peuvent survenir, modifiant ainsi le pronostic. Le but de notre travail était de décrire les aspects cliniques et thérapeutiques de 15 cas de rickettsiose avec atteinte encéphalitique.

## Méthodes

Étude prospective concernant 15 patients atteints d’une encéphalite aiguë à *Rickettsia conorii,* pris en charge entre 2015 et 2018. Tous les cas ont été confirmés par PCR et par immunofluorescence indirecte (IFI). Les preuves radiologiques (IRM cérébrale) et électriques (EEG) ont été établies pour les 15 patients.

## Résultats

L’âge moyen était de 37 ± 7,8 ans, 13 étaient des hommes (86.7 %). Les céphalées étaient retrouvées dans 100 % des cas, troubles de la conscience dans 53.3 % (n = 8), crises épileptiques dans 26.7 % (n = 4) et ataxie cérébelleuse dans 20 % (n = 3). L’EEG avait objectivé des signes de souffrance cérébrale diffuse dans 53,3 % (n = 11) et l’IRM cérébrale des lésions évocatrices d’une encéphalite dans 26,7 % (n = 4). La PCR et l’IFI ont confirmé l’infection à *Rickettsia conorii* dans 100 % des cas. Le traitement était à base de cyclines et fluoroquinolones. L’évolution était favorable dans 14 cas (93,3 %) et fatale dans un cas.

## Discussion

Des complications systémiques sont observées dans les rickettsioses, parmi lesquelles les complications neurologiques. L’encéphalite est rarement rapportée. Le mécanisme prépondérant est une vascularite. L’encéphalite s’observait avec une grande fréquence au cours de la deuxième semaine suivant le début de l’infection. Elle était caractérisée par l’important polymorphisme de son expression. Le traitement de référence est la doxycycline. La place des fluoroquinolones dans le traitement des rickettsioses constitue un sujet de débat.

## Conclusion

Le diagnostic de rickettsioses doit être évoqué au cours de manifestations neurologiques évoluant dans un contexte fébrile, qu’il s’agisse d’encéphalite ou de méningo-encéphalite.

# Research article: Recherche des patients vivants avec le VIH perdus de vue au CTA de Donka : expérience de la Fondation espoir de Guinée

**Published:** 2024-06-07

**Authors:** Aïssatou Bah LAMANARA, Joseph BALATH, Diallo AÏSSATOU, Lamarana BAILO, Brigitte VAKOU SINGA, Kanté Mamadou DJOULDE, Moussa SAAVNÉ, Mohamed CISSE

**Affiliations:** Fondation Espoir de Guinée, CHU de Donka Conakry, Guinée

## Introduction

Malgré la multiplicité des sites de prise en charge des personnes vivant avec le VIH (PVVIH) et la gratuité du traitement antirétroviral en Guinée, la prise à vie de la thérapie antirétrovirale (TARV) engendre souvent la lassitude, le découragement responsable d’une augmentation de la proportion de patients perdus de vue (PDV).

L’objectif de notre travail était de rapporter notre expérience dans la recherche des patients perdus de vue suivis au centre de traitement ambulatoire de Donka.

## Matériels et méthodes

Il s’agissait d’une étude transversale, descriptive sur une période de 24 mois allant de janvier 2021 à décembre 2022. Nous avons inclus dans cette étude, tous les patients sous traitements ARV n’ayant pas donné de nouvelles trois mois après la date de leur dernier rendez-vous. Pour chaque patient, nous avons recherché les motivations de l’abandon du traitement par appel téléphonique.

## Résultats

Durant la période d’étude, nous avons identifié 785 patients ayant raté au moins trois rendez-vous pour la dotation en ARV. Parmi lesquels 437 (55,6 %) patients, ont accepté la réinitiation au TARV, 22 à cause des coûts élevé des examens biologiques, 25 transférés sortant officiel, 92 patients injoignables parmi lesquels 32 avait le numéro de l’opérateur Sotelgui qui est en arrêt de fonctionnement, 17 faux numéros mentionnés dans les dossiers ARV, 24 décédés. Nous avons identifié 149 (18,9 %) patients ayant promis de revenir pour une réinitiation du TARV et 19 patients ayant refusé de collaborer au téléphone parmi lesquels neuf patients estimaient être guéris de l’infection par le VIH.

## Conclusion

Les PVVIH perdus de vue impactent négativement l’atteinte des objectifs « 3 x 95 » de l’ONUSIDA pour mettre fin à l’épidémie. Les facteurs associés doivent être identifiés dans une étude ultérieure afin de réduire la prévalence des PDV au sein de notre cohorte.

# Research article: Les représentations des vaccins pédiatriques et contre le Covid-19 au Niger : des enseignements pour une communication adaptée

**Published:** 2024-06-07

**Authors:** Bernard SEYTRE, Chaibou SANOUSSI

**Affiliations:** bnscommunication, 7 rue Ledion, 75014 Paris

Dans le cadre du programme AFROVAX de renforcement de la vaccination mené par la Croix-Rouge Française, nous avons mené au Niger une enquête socio-anthropologique mixte destinée à comprendre les représentations de la population sur la vaccination, afin de fournir les bases à une stratégie de communication pour promouvoir les vaccins pédiatriques et contre le Covid-19.

Pour l’enquête quantitative, nous avons interrogé 509 personnes de Niamey et de 2 villages de la région de Zinder, en abordant séquentiellement la vaccination en général, la vaccination pédiatrique, le Covid-19 et la vaccination contre le Covid-19. Pour le volet qualitatif, nous avons réuni quatre groupes focus et mené 10 entretiens individuels, dans les mêmes localités.

Nos résultats montrent une adhésion quasiunanime à la vaccination pédiatrique. La crainte des effets secondaires, mentionnés par 30,6 % des enquêtés, un manque d’amabilité du personnel de santé et le temps d’attente, mentionnés respectivement par 21,4 % et 16,9 %, apparaissent comme des facteurs contribuant à expliquer une déperdition après les premières doses et un manque de respect du calendrier vaccinal.

La faible adhésion à la vaccination contre le Covid-19 s’explique par la conjonction de plusieurs idées erronées sur la pandémie. D’abord, la majorité de la population pense que la maladie n’est pas présente dans le pays, 43,8 % seulement estimant qu’elle l’est. Ensuite, la quasi-totalité de la population pense que seuls des personnes malades transmettent le virus responsable du Covid-19 et 12,8 % seulement estiment connaître quelqu’un qui a eu la maladie, 5,1 % dans leur entourage. L’ensemble de ces représentations se traduit par une perception très faible du risque de contracter la maladie.

Nos résultats indiquent aussi que les rumeurs négatives qui circulent sur les vaccins pédiatriques et contre le Covid-19 ne semblent pas jouer de rôle significatif.

# Research article: Connaissances, attitudes et pratiques des praticiens du Service de pathologie et chirurgie buccales du CHU de Tlemcen visà-vis du Covid-19

**Published:** 2024-06-07

**Authors:** Samira BENBEKTI ABDREBBI, Amine MESLI, Ilyes TCHOUAR, Manal BACHADEL, Manal FIZAZI, Suzane CHABANI ASSALA

**Affiliations:** Faculté de médecine, Université Abou Bekr Belkaid de Tlemcen, Algérie; Service d’épidémiologie et de médecine préventive, Centre hospitalo-universitaire de Tlemcen, Algérie

## Introduction

Le personnel soignant représente depuis le début de la pandémie de la Covid-19 le corps professionnel le plus exposé à l’infection virale.

## Objectif

Évaluer les connaissances, attitudes et pratiques (CAP) chez le personnel de santé bucco-dentaire vis-à-vis de la Covid-19.

## Matériel et méthodes

Étude descriptive transversale réalisée en 2022. La population de l’étude était représentée par les praticiens du service de Pathologie et Chirurgie Buccales du CHU de Tlemcen.

## Résultats

L’enquête a concerné 38 sujets. Une légère prédominance féminine a été notée (sex-ratio : 0,81). La tranche d’âge la plus représentée était celle comprise entre 20 et 25 ans, qui est représentée majoritairement par les internes du Service de pathologie et chirurgie buccales. Les sujets enquêtés ont utilisé différentes sources d’informations pour se renseigner sur la Covid-19 dont 32 % se sont référés à travers des professionnels de santé, 26 % ont utilisé les réseaux sociaux et les médias et 12 % par l’intermédiaire de leur famille et amis. Presque 80 % des praticiens connaissent le nom du virus responsable de la maladie. Les connaissances sur le mode de transmission étaient suffisantes (80 %) et sur les symptômes étaient moyennes (77 %). La totalité des praticiens déclare que même si une personne est asymptomatique, elle peut être contagieuse.

La totalité du personnel enquêté trouve que la nature de leur pratique les expose à un risque élevé d’infections croisées en raison de leur exposition à des micro-organismes pathogènes et à des virus provenant de la cavité buccale et des voies respiratoires des patients. Plus de la moitié des praticiens n’ont jamais demandé un test PCR avant les interventions chirurgicales. La majorité des praticiens utilisent les équipements de protection individuelle (EPI) durant les interventions chirurgicales dont les plus utilisés sont : sur blouses, gants, masque chirurgical ou bavette FFP2/KN95 et lunettes de protection et ou visière. La majorité des praticiens change de gants entre deux patients successifs. Le lavage chirurgical des mains avant les actes chirurgicaux n’est réalisé que chez 71 % des praticiens; 61 % des praticiens déclarent avoir respecté l’ordre de l’habillage des équipements de protection individuelle.

## Conclusion

Bien que le niveau de connaissances et d’attitudes concernant la maladie soit acceptable chez le personnel de santé bucco-dentaire du CHU de Tlemcen, il est indispensable d’assurer des actions visant à améliorer leurs connaissances et changer leurs comportements, afin de lutter contre cette maladie et faire face à une éventuelle épidémie à transmission respiratoire.

# Research article: *Success Story* de la gestion de la 2^e^ épidémie d'Ebola en Guinée

**Published:** 2024-06-07

**Authors:** Ibrahima BAH, Jacques NGAMBONGO MOPILI, Sophie BOKATA, Hyppolyte SITUAKIBANZA, Thaddée ODIO

**Affiliations:** Conakry, Guinée

En février 2021, la Guinée a connu sa 2^e^ épidémie de la maladie à virus Ebola (MVE) après celle de 2014-2016 qui avait occasionné 2 544 décès parmi les 3 814 cas enregistrés. Cette épidémie de l’année 2021 notifiée dans une des 10 sous-préfectures de N’Zérekoré située à 42 km du chef-lieu a enregistré 16 cas confirmés dont 5 décès, soit un taux de létalité de 31,2 %.

Contrairement à la précédente épidémie (2014-2016), cette dernière a été circonscrite et contrôlée en seulement trois mois évitant ainsi la propagation de la maladie à d’autres localités du pays et dans la sous-région ouest africaine. Ce succès a été rendu possible grâce au renforcement de la surveillance épidémiologique et à la mise en œuvre des activités de riposte dans les premières heures qui ont suivi la confirmation du premier cas avec l’appui des partenaires techniques et financiers impliqués dans la gestion des urgences de santé en Guinée. Parmi ces facteurs de succès nous avons :

- Coordination : le fort leadership et l’expérience de l’ANSS dans la gestion des urgences de santé publique notamment pour les maladies à potentielle épidémique (MPE) comme le cas de la MVE avec la décentralisation de la coordination opérationnelle de la riposte à travers les COU-SP régionaux et préfectoraux. Durant la période de l’épidémie, des réunions régulières de concertation et de suivi des activités de la riposte se tenaient régulièrement (2 fois/semaine) et en ligne à travers les équipements de visio-conférence acquis pour la riposte contre le Covid-19.
- Investigation/riposte : la mise en place et l’opérationnalisation des équipes d’intervention rapide avec les équipes préfectorales d’alerte et de riposte aux épidémies (EPARE) ont permis la détection précoce des cas et la circonscription du foyer épidémiologique avec l’appui des agents de la surveillance à base communautaire (SBC). Ces équipes préfectorales ont été mises à contribution dans l’investigation et la riposte par l’équipe régionale d’alerte et de réponse aux épidémies de la région (ERARE) de N’Zérekoré et par une équipe nationale en provenance de l’ANSS et des partenaires (OMS, AFENET…). À noter que toutes ces équipes (EPARE et ERARE) ont été formées en épidémiologie d’intervention par le programme de FELTP.
- Prise en charge : la disponibilité et la fonctionnalité d’un centre de traitement épidémiologique (CT-Epi) dans toutes les préfectures du pays a permis la prise en charge immédiate et sur place des cas avec un personnel formé et équipé pour assurer une meilleure prise en charge des cas de MPE. Cette prise en charge était médicale, psychologique et alimentaire.
- Laboratoire : contrairement à l’épidémie de 2024, le pays dispose de nos jours d’un réseau de laboratoires pour le diagnostic des MVE avec une cartographie de neuf laboratoires opérationnels. Pour cette épidémie, quatre laboratoires ont été mis à contribution selon la cartographie des cas recensés.
- Immunisation : il y a eu également des innovations dans la lutte contre Ebola, notamment la vaccination en ceinture des personnes à risque autour de chaque cas et l’utilisation de nouvelles molécules dans le traitement des malades qui ont été déterminantes pour réduire la létalité.

Ainsi la vaccination en ceinture autour des cas confirmés et probables du 23 février au 30 avril 2021 a permis de vacciner 8 255 personnes dont 936 contacts, 5 013 contacts des contacts et autres, et 2 306 travailleurs de première ligne (personnel médical, agents de santé publique …)

La communication et l’engagement communautaire : la mise à contribution des radios communautaires, des plateformes communautaires « Une seule Santé », des ressortissants des villages des localités touchées et l’implication des socio-anthropologues a facilité l’adhésion et l’engagement communautaire aux activités de riposte.

Enfin, il faut noter la synergie d’actions des partenaires qui ont apporté un appui technique et financier au ministère de la Santé pour une riposte plus efficace et coordonner.

# Research article: Évaluation de la recrudescence des cas de paludisme chez l'adulte au CHR Georges Rawiri de Lambaréné (CHRGRL) Gabon

**Published:** 2024-06-07

**Authors:** Madiou DIALLO, Roxana Rodriguez PEREZ, Davy Tanguy MENDOME ABESSOLO

**Affiliations:** Service Maladies Tropicales CHRGRL, Lambaréné, Gabon

Après une suspension de près de 10 ans (2012-2022) de l’appui par le Fonds mondial, le Gabon bénéficiera de nouveau d’une aide du Fonds mondial avec un accent mis par le pays sur la distribution de moustiquaires imprégnées. Le CHR de Lambaréné au centre du pays, non loin de l’équateur, comporte huit services dont un de MIT (pour les plus de 15 ans) et 129 lits. Devant l’augmentation observée des cas de paludisme grave, nous avons entrepris une étude comparant les données du paludisme en 2019 (n = 209) et 2022 (n = 606).

## Résultats

Le pourcentage des cas de paludisme hospitalisés par rapport au nombre total des hospitalisés est passé de 43 à 74 %. Les cas sont plus nombreux dans le genre masculin et chez les moins de 50 ans. Certains comportements sont analysés dont le parcours de soin avant hospitalisation. Le taux de guérison (TDR ou GE négatifs) est passé de 92 % à 88 %. Les causes de ce changement sont discutées et des études spécifiques sont envisagées.

# Research article: La primaquine revisitée dans les pays impaludés par la mise à disposition de formes pédiatriques adaptées et une implémentation rationnelle au Burkina Faso, Burundi, Éthiopie, et Madagascar

**Published:** 2024-06-07

**Authors:** Pascal MILLET, Bob TAYLOR, Didier MENARD, Julie Ngoc NGUYEN, Héléna PRADO, Thoopmanee KAENDIAO, Teferi MEKONNEN, Sodiomon B. SIRIMA, Arsène RATSIMBASOA, Joseph NYANDWI, Sheila VARADAN

**Affiliations:** ReMeD, 21bis Avenue du Commandant l’Herminier, 44600 Saint Nazaire, France

La primaquine est un médicament essentiel utilisé en traitement curatif de *Plasmodium vivax* et *ovale* et en traitement préventif comme gamétocytocide pour réduire le risque de transmission de *P. falciparum.* Or il n’existe pas de formes pédiatriques de primaquine respectant les standards internationaux de qualité. Pour pallier ce manque, deux projets internationaux ont été financés par le partenariat Europe-Pays en développement pour les essais cliniques (EDCTP). Le premier projet, DPP, financé de 2020 à 2024, est porté par l’Université d’Oxford et le Réseau médicaments et développement (ReMeD) et a pour objectif le développement de formulations pédiatriques de primaquine adaptées à l’âge et au poids. L’objectif est de donner accès sur le terrain à la primaquine à des dosages pédiatriques répondant aux normes internationales de qualité, d’innocuité et d’efficacité, et à globaliser l’utilisation de traitement du paludisme à base de primaquine. Pour cela, un consortium euro-africain (Éthiopie, Burkina Faso, France et Royaume-Uni/Thaïlande) et un partenariat avec des industriels indiens (IPCA et Bilcare) a été mis en place. Ce premier projet ira jusqu’à la préqualitication par l’OMS. Le second projet IMPRIMA, financé de 2023 à 2027, est porté par l’Université de Strasbourg et le réseau ReMeD, et a pour objectif l’implémentation de la primaquine à dose faible pour limiter la transmission de *P. falciparum* dans les pays qui présentent des zones de préélimination du paludisme. Ce projet se fera en partenariat avec les programmes de lutte contre le paludisme et les autorités de santé de Madagascar, Burkina Faso et Burundi. Les résultats de l’étude DPP seront présentés, ainsi que la stratégie globale autour de l’usage rationnel de la primaquine et les enjeux sur le terrain auprès des professionnels de santé et de la population.

Liste des participants aux deux consortia : AHRI: Armauer Hansen Research Institute, Addis Ababa, Ethiopie; Bilcare : Entreprise pharmaceutique de packaging, Pune, Inde; Centre national d’application de recherches pharmaceutiques, Antananarivo, Madagascar; ClinSearch : Prestataire de EBI pour la modélisation de l’acceptabilité des médicaments, France; EBI : École de biologie industrielle, Cergy, France; Institut National de santé publique, Bujumbura, Burundi; IPCA : The Indian Pharmaceutical Combine Association Limited, Mumbai, Inde; Groupe de recherche actions en santé (GRAS), Ouagadougou, Burkina Faso; Leiden Law School - African Studies Centre Leiden, The Nehterlands; LTPIB : Laboratoire de technologie pharmaceutique industrielle de Bordeaux, France; MORU : Mahidol-Oxford Research Unit, Bangkok, Thaïlande; NDM : Nuffield Department of Medicine - Centre for Trop. Medicine & Global Health, Oxford, Royaume-Uni; ReMeD : Réseau Médicament & Développement; UCL : Université de Strasbourg, France; University College of London, Royaume-Uni.

# Research article: Le suivi de la santé des personnels civils travaillant dans les emprises militaires françaises au Sud

**Published:** 2024-06-07

**Authors:** Jean-François FERRAND

**Affiliations:** Professeur agrégé du Val de Grâce, Service de santé des armées

Les emprises militaires au Sud ne sont pas homogènes. Elles comprennent des forces de souveraineté dans des départements et collectivités d’outre-mer, des forces de présence comme à Djibouti dans un environnement de travail stabilisé et des situations comme les opérations extérieures ou les conditions de travail des militaires ou des personnels civils de recrutement local peuvent être difficiles du fait du contexte opérationnel. Logiquement, le suivi de la santé des personnels civils de recrutement local doit être effectué, sauf exception, par les structures locales de médecine du travail. L’absence de prestataires conduit parfois à ce que ce suivi soit assuré dans le cadre de la médecine de prévention par les équipes du service de santé des armées.

Plus spécifiquement, le suivi des personnels civils de recrutement local affecté à la manipulation des denrées alimentaires a souvent été l’objet de préoccupations afin d’éviter la survenue de toxi-infections alimentaires collectives. Pour améliorer et homogénéiser les pratiques la direction centrale du service de santé des armées a diffusé des conduites à tenir.

Quels que soient les environnements professionnels, les équipes en charge de ce suivi doivent avoir pour objectif la préservation de l’état de santé, ce qui nécessite aussi une implication dans l’évaluation et la prévention des risques en milieu de travail. L’efficacité des actions de ces équipes dépend de leurs connaissances dans le champ de la santé au travail.

Idéalement, le suivi de la santé des personnels civils de recrutement local doit être clarifié lors de l’élaboration ou de la révision des accords et traités. Dans tous les cas, l’implication des équipes du service de santé des armées et les conseils délivrés au commandement bénéficieront aux militaires dans le cadre de la protection santé de la force et aux personnels civils de recrutement local du fait de l’amélioration de l’hygiène et des conditions de travail au sein des emprises militaires françaises au Sud.

# Research article: Règles et leurs obligations de travail dans la pêche sousmarine familiale au Japon

**Published:** 2024-06-07

**Authors:** Yoshitaka MORIMATSU, Kiyotaka KOHSHI, Hideki TAMAKI, Yukio MURATA, Toru MOCHIZUKI, Tatsuya ISHITAKE

**Affiliations:** Cours de médecine environnementale, Université Kurume - 64, Asahi-machi Kurume city, Fukuoka, 830-0011, Japon

La pêche en plongée est populaire au Japon depuis longtemps et il existe deux types de pêche en plongée, l’une est la pêche en plongée libre dans laquelle le souffle est retenu pendant la plongée, et l’autre est la pêche en plongée à l’aide d’un dispositif d’alimentation en air. Le « Funado » est une méthode de pêche dans laquelle un couple, parents et enfants, etc. monte à bord d’un bateau en tant qu’unité familiale et utilise une poulie pour remonter le partenaire qui plonge immédiatement à l’aide d’un poids. La région de Hagi de la préfecture de Yamaguchi est une zone où les hommes pratiquent la pêche en apnée. En conséquence, les accidents de décompression se produisent fréquemment et notre laboratoire a signalé un accident de décompression dû à la plongée sous-marine pour la première fois au monde. De plus, une enquête par questionnaire menée chez ces pécheurs a révélé que les facteurs de risque d’accident de décompression étaient l’obésité, le diabète, le temps passé sur les fonds marins et le temps passé à se reposer à la surface de la mer. Après cela, une formation à la sécurité leur a été dispensée et aucun décès dû à un accident de décompression n’a été observé dans cette zone. La pêche sous-marine à adduction d’air, qui utilise un appareil à adduction d’air, est également pratiquée famille par famille. En 2019, deux des trois frères et leur fils qui pratiquaient la pêche sous-marine avec alimentation en air sont morts d’une intoxication au monoxyde de carbone en raison d’un mauvais entretien du système d’alimentation en air. De plus, trois plongeurs travaillant dans une pisciculture de la préfecture de Kagoshima sont morts au cours des cinq dernières années. Actuellement, il n’y a pas de normes légales pour l’inspection des équipements d’alimentation en air installés sur les navires, de sorte que l’état de maintenance des équipements d’alimentation en air dépend de son propriétaire. De plus, comme les plongeurs qui sont seuls maîtres ne peuvent pas s’affilier à l’assurance contre les accidents du travail, la plupart de leurs décès au travail sont traités comme des décès pour cause de maladie. La pêche en plongée est dangereuse, elle est donc pratiquée dans une relation de confiance avec les membres de la famille, mais il est nécessaire d’élaborer des lois et d’assurer la responsabilité de la gestion de la sécurité des familles à qui leur vie est confiée.

# Research article: Accidents de travail chez les femmes mareyeuses du port de pêche de Cotonou, Bénin, 2022

**Published:** 2024-06-07

**Authors:** Mênonli ADJOBIMEY, Gilles Gbede EBILIZER, Vikkey HINSON

**Affiliations:** Unité de recherche et d’enseignement en santé au travail et environnement FSS Cotonou, Bénin

## Introduction

Les accidents de travail (AT) constituent les risques professionnels les plus fréquents dans certains corps de métiers. Les mareyeuses paraissent parmi les plus touchées. L’objectif de la présente étude est de déterminer la prévalence et les facteurs associés aux AT chez les mareyeuses.

## Méthodes

Il s’est agi d’une étude transversale descriptive et analytique. La taille de l’échantillon calculée était de 355 mareyeuses. Il a été procédé à un échantillonnage non probabiliste. Une enquête par questionnaire a été effectuée. Il a été procédé à une analyse descriptive par calcul des proportions et des moyennes puis à une analyse bivariée et multivariée. Des tests de Chi2 et de Fisher ont été réalisés au seuil de signification de 5 %. Des odds ratios ont été calculés comme mesure d’association. L’approbation du comité d’éthique a été obtenue ainsi que l’autorisation du responsable de l’Union nationale des pêcheurs marins artisans et assimilés du Bénin.

## Résultats

Au total, 374 mareyeuses d’une moyenne d’âge de 43,7 ± 11,7 ans et une ancienneté moyenne au poste de 21,8 ± 13 ans ont été incluses. La fréquence des AT sur site était de 86,4 %. Les causes des AT selon les enquêtées étaient : manque d’EPI (78,9 %), défaut de concentration (46,7 %), outils non adéquats (14,6 %), sol glissant (9,9 %) et autres (8,7 %). Les facteurs associés aux AT étaient une perception négative de l’avenir professionnel (p<0,001; ORa = 3,74, IC_95_ % [1,98 - 7,25]) et une faible perception de contrôle de la mareyeuse sur l’activité (p = 0,025; ORa = 2,29, IC_95_ % [1,11 - 5,11])

## Conclusion

Les AT sont très fréquents chez les mareyeuses et les facteurs associés sont modifiables. Des stratégies de prévention liées à la réorganisation du travail pourront aider à une meilleure prévention des AT.

# Research article: Prévalence et facteurs associés au burnout chez les enseignants des cours secondaires de la ville de Cotonou au Benin en 2022

**Published:** 2024-06-07

**Authors:** Mênonli ADJOBIMEY, Jolianelle Dassi Gbenou Ranty, Antoine Vikkey Hinson

**Affiliations:** Unité de recherche et d’enseignement en santé au travail et environnement FSS Cotonou, Bénin

## Introduction

La pénibilité psychologique du métier d’enseignement entretient un stress chronique qui évolue inéluctablement vers un burnout (BO) en absence de mesures préventives adéquates. L’objectif de cette étude est de déterminer la prévalence et les facteurs associés au BO chez les enseignants des cours secondaires de la ville de Cotonou en 2022.

## Méthodes

Il s’est agi d’une étude transversale descriptive à visée analytique qui s’est déroulée du 19 juillet au 19 août et du 1^er^ octobre au 31 octobre 2022. L’échantillonnage a été fait selon une technique de sondage aléatoire à trois degrés. Les questionnaires de Maslach sur le BO et celui de Karasek sur le stress professionnel ont été utilisés. L’analyse des données a été réalisée à l’aide du logiciel R 4.1.1. Après une analyse descriptive, une analyse multivariée par régression logistique binaire a permis l’identification des facteurs associés. Le seuil de significativité choisi était de 5 %.

## Résultats

Au total, 270 enseignants ont été inclus dans l’étude, dont 208 (77,00 %) étaient des hommes. L’âge moyen des travailleurs était de 38,03 ans ± 8,30 ans. Selon le Maslach Burnout Inventory, 58,51 % des enseignants présentaient un BO avec 41,10 % d’enseignants en épuisement émotionnel, 27,40 % en dépersonnalisation et 21,90 % en perte d’accomplissement personnel. La sévérité du BO était de faible à modérée chez presque tous les enseignants. La survenue du BO était associée à la non pratique de sport (p = 0,003; ORa = 2,38 [1,32; 4,28]), le stress professionnel (p = 0,036; ORa = 1,86 [1,04; 3,34]) et un déséquilibre effort/récompense (p< 0,001; ORa = 5,98 [2,24; 15,98]).

## Conclusion

La prévalence du BO est élevée chez les enseignants du secondaire de la ville de Cotonou. Une étude à une plus grande échelle incluant la recherche de ses conséquences sur l’enseignant et l’apprenant s’impose afin de pouvoir vite agir pour la mise en œuvre d’un programme de prévention.

# Research article: Secteurs professionnels et couverture sanitaire à Madagascar : impact sur la grossesse

**Published:** 2024-06-07

**Authors:** Zoly RAKOTOMALALA NIVOARIMELINA, Jean Tsindrame Soanjara, Patrick Marco Ramarokoto Malalafinaritra, Gabriel Randaoharison Pierana, H. Rakotomahenina

**Affiliations:** Centre hospitalier universitaire Androva, Faculté de Médecine de Mahajanga, Mahajanga, Madagascar

Madagascar est un pays à faible revenu avec un salaire minimum estimé à 250 000 ariary soit 50 euros par mois. En outre, une différence conséquente de la couverture médicale est constatée entre les secteurs informel et formel. Ceci nous incite à évaluer la relation entre la prise en charge sanitaire et la survenue de pathologies obstétricales et périnatales.

Pour cela, nous avons mené une étude sur les femmes enceintes admises au Complexe mère-enfant de l’Hôpital Androva à Mahajanga, Madagascar, concernant le déroulement et l’issue de leur grossesse en fonction de leur statut professionnel.

Le résultat préliminaire sur deux mois (mars-avril 2023), nous a permis de recruter 160 femmes enceintes admises dans notre service dont 88 (55 %) travaillaient et 108 (90 %) bénéficiaient du revenu de leur conjoint. Seules 28 femmes (17,50 %) avaient bénéficié d’un remboursement total ou partiel de leurs frais médicaux par leur entreprise ou par celle de leur conjoint. Le gain mensuel du foyer était inférieur à 500 000 ariary (100 euros) dans 41,7 % des cas. Ce revenu ne présentait pas un lien significatif avec les complications obstétricales. L’absence de couverture médicale par leur entreprise ou celle du conjoint constituait un facteur protecteur à la survenue de fausses couches spontanées (p = 0,02, RR=0,11), d’une prééclampsie (p = 0,000001, RR = 0,02) et de décès périnataux (p = 0,038, RR = 0,21).

La grande majorité de la population fait partie du secteur informel, donc ne disposant pas encore de couverture sanitaire. Par ailleurs, les femmes ayant une prise en charge des frais médicaux auraient tendance à négliger leur santé durant la grossesse et ne se prépareraient pas financièrement aux pathologies obstétricales qui sont souvent fréquentes et inattendues chez les femmes qui travaillent à Madagascar.

# Research article: Analyse des risques auditif et respiratoire dans plusieurs branches d'activités du secteur informel à Cotonou

**Published:** 2024-06-07

**Authors:** Mênonli ADJOBIMEY

**Affiliations:** Unité de recherche et d’enseignement en santé au travail et environnement FSS Cotonou, Bénin

Le secteur informel contribue pour une part importante à l’économie des pays en voie de développement. Au Bénin, il représente près de 80 % des travailleurs. Malgré le nombre important de travailleurs concernés, il n’existe presque pas de politique de santé sécurité au travail et les conditions de travail sont précaires avec une absence quasi- totale de moyens de prévention technique collective et individuelle. Une analyse des postes de travail dans plusieurs catégories d’activités a montré des niveaux d’exposition élevés. Au plan auditif, un niveau de bruit supérieur à 90 dB a été observé chez des ferblantiers, des meuniers de céréales du marché Dantokpa, des soudeurs et des travailleurs de scieries. Des niveaux de poussières supérieures à la norme ont été retrouvés chez les meuniers de céréales, les utilisateurs de meules de mur sans aucun équipement de protection individuelle. Les prévalences des troubles auditifs et respiratoires ont varié selon les branches d’activités. La connaissance des travailleurs sur les risques sanitaires encourus ainsi que les moyens de prévention nécessaires était faible dans presque toutes les branches d’activités. Un accompagnement du secteur informel par les politiques publiques est nécessaire pour améliorer les conditions de travail et de vie de la majorité des travailleurs concernés.

# Research article: Les facteurs extra professionnels et leurs impacts sur la santé au travail dans les pays du sud : l'exemple du Sénégal

**Published:** 2024-06-07

**Authors:** Mor NDIAYE

**Affiliations:** Service de médecine du travail, FMPO, UCAD, Dakar, Sénégal

Le travailleur appartient à une famille, une entreprise et évolue au sein d’un environnement social et d’une communauté.

L’absence et/ou la méconnaissance des textes législatifs et réglementaires en matière de santé au travail, la récession économique, l’économie informelle, l’importance et la sous qualification de la main d’œuvre, la mondialisation, les croyances religieuses, le mode de vie, le niveau d’instruction, les priorités de la santé publique, la nutrition, le sommeil, l’activité physique, la pratique sportive et les conduites addictives (tabac, alcool, drogue) constituent les facteurs extra professionnels qui agissent sur la santé au travail.

Dans nos pays, ces facteurs ont un impact certain sur la promotion de la santé au travail, l’accroissement des risques professionnels, la sous déclaration des accidents de travail et maladies professionnelles, l’usure prématurée au travail, la survenue de stress au travail, l’émergence de maladies métaboliques, la détérioration des conditions de travail, les conflits sociaux, l’absence voire l’inefficacité de l’éducation ouvrière.

# Research article: À propos des cas de personnel soignant contaminés par la Covid-19 des établissements de soins de Tlemcen

**Published:** 2024-06-07

**Authors:** Meziane ZAKIA, Taleb ABDESLAM

**Affiliations:** Université Abou Bakr Belkaid, Faculté de médecine, Tlemcen, Algérie

## Introduction

La pandémie par le SARSCoV-2 a touché le monde entier, néanmoins certaines populations sont à haut risque de contamination, c'est le cas du personnel de santé qui constitue la première barrière face à cette pandémie.

## Objectifs

Décrire les caractéristiques cliniques, biologiques du personnel contaminé.

## Matériels et méthodes

Il s’agit d’une étude descriptive, allant sur une période de neuf mois, d’août 2020 jusqu’à avril 2021 comportant le personnel de la santé travaillant dans le CHU et EHS TLEMCEN qui se s’est présenté au service de médecine du travail (unité Covid-19) infecté par la Covid-19 pour une consultation. L’ensemble des sujets enquêtés a été soumis à un questionnaire.

## Résultats

Au total, 521 soignants diagnostiqués Covid-19 positifs ont été enregistrés, soit une proportion de 12 %. Le diagnostic de la Covid-19 a été confirmé chez 425 (81,6 %) patients par RT-PCR, et 149 (28,6 %) patients par TDM thoracique sans injection. L’âge moyen est de 36 ans +/- 9 ans avec une nette prédominance féminine avec un taux de 72 % de femmes.

Une évolution croissante des cas de contamination du personnel a été observée de juin à novembre 2020 avec un pic en juillet et en novembre suivi d’une décroissance à compter de décembre 2020 pour aboutir à 04 cas en avril 2021.

L'infection a été observée dans le service de gynécologie et les UMC, ceci pourrait être expliqué par le fait qu'il comportait l'effectif le plus important en personnel, les rapports étroits entre les agents hospitaliers partageant les mêmes locaux avec parfois une difficulté de maintenir une distanciation correcte. Le poste le plus touché était les ISP avec un effectif de 16 % suivi par les médecins résidant 14 % suivi par les ATS 12 %.

Les symptômes les plus retrouvés sont respectivement asthénie 413, anosmie 338, agueusie 293, fièvre 288, courbature 247, céphalée 255. Les groupes sanguins les plus retrouvés sont respectivement le groupe O et le groupe A

## Conclusion

Lors de la crise sanitaire de la Covid-19, les hôpitaux en Algérie ont été très impactés, en particulier le CHU de Tlemcen qui a été désigné Hôpital Covid-19.

Le service de la médecine du travail du CHU de Tlemcen s’est organisé pour permettre le dépistage, le suivi des nouveaux cas de Covid. Notre priorité est d’informer et sensibiliser notre personnel sur l’intérêt du port des moyens de protection individuelle, les mesures de distanciation et surtout la vaccination.

# Research article: Bonne santé et bien-être au travail et contribution des cellules focales de lutte contre le sida

**Published:** 2024-06-07

**Authors:** Solange AMETHIER, Baba SANGARE, Christine Mariette Alida BAKOU NIANGORAN, Alexandre KOFFI KOUAMÉ

**Affiliations:** Cellule médico-sociale du ministère de la Femme, de la Famille et de l’Enfant, Abidjan, Côte d’Ivoire

Le ministère de la Femme, de la Famille et de l’Enfant a mis en place des cellules focales de lutte contre le sida à travers sa Cellule médicosociale dans les directions, programmes et structures socio-éducatives de la Direction Régionale des lagunes avec l’appui du Programme national de lutte contre le Sida et de l’ONU sida. Dans le cadre de la mise en œuvre de la réponse multisectorielle en vue de réduire l’impact du VIH/sida sur les populations et en milieu du travail, 36 cellules focales ont été installées. Pour mener à bien les activités, 36 points focaux ont vu leur capacité renforcée sur les techniques de communication pour le changement social et de comportement. Les activités menées par les cellules focales suite aux causeries éducatives de groupe et individuelle portant sur les thématiques telles que les généralités sur les IST et le VIH, la prévention des IST et du VIH, les services de dépistage VIH, le traitement ARV, genre et VIH, la promotion de l’utilisation du préservatif et la référence pour Prep ont permis de toucher 1 725 travailleurs, 5 175 usagers des services socioéducatifs, de réaliser le test de dépistage de 345 personnes et de référer 37 personnes de janvier à décembre 2022. Les activités de coordination à travers les supervisions effectuées ont permis de relever la persistance de la stigmatisation, la non-utilisation et ou la mauvaise utilisation du préservatif, l’insuffisance de gadgets et supports de sensibilisation, la sous notification dans l’enregistrement des données sur les fiches d’activités.

# Research article: Partage de pratiques professionnelles en santé au travail

**Published:** 2024-06-07

**Authors:** Claire FABIN

**Affiliations:** ASSITEB-BIORIF 33 rue de Metz - 94170 Le Perreux, France

Depuis plus de 10 ans, participation à des congrès africains de sécurité-santé au travail et partage de pratiques professionnelles de terrain acquises en région parisienne.-

## Objectif

À partir de situations de travail et pathologies chroniques rencontrées, montrer l’importance de l’étude du poste de travail, des outils d’aide et référentiels pour évaluer les risques et les maîtriser.

## Méthodes

Présentations, lors de congrès en santé au travail en Afrique subsaharienne, Cotonou (2012 - 2016), Dakar (2018), Douala (2016 - 2019), Grand Bassam (2017) d’outils pour collecter des données : étiquetage des produits chimiques, fiches de données de sécurité, outils de mesurage d’ambiances, notamment chimiques, sonores, thermiques et des vibrations, outils de repérage et d’évaluation des postures, notamment rachidiennes et membre supérieur, des ports de charges et des troubles musculosquelettiques, démarche ergonomique en entreprise.

## Résultats

Trois types d’exemples sont présentés.

- Risques professionnels : risques chimiques (mise en place de fiches : repérage des produits, recueil d’exposition, suivi post professionnel, traçabilité professionnelle, *cursus laboris,* démarche d’évaluation notamment pour cancérogènes, mutagènes et reprotoxiques, démarche de prévention voire de substitution); risque vibrations corps entier (présentation d’études ergonomiques et épidémiologiques, réduction des pathologies en agissant sur la durée de conduite, réglage du siège, mode de travail ou état du sol, méthodes simplifiées d’évaluation permettant d’initier un plan de prévention en absence de métrologie de terrain); présentation d’études sur les apports de l’information formalisée pour réduire l’exposition au bruit, vibrations et risques chimiques.
- Situations de travail : exemples et outils d’aide à l’évaluation et réduction des manutentions, postures et TMS aux postes d’infirmière, secrétaire, ouvrier et technicien de laboratoire; dans le transport routier, contraintes et démarches d’amélioration pour les manutentions, manœuvres et contraintes technico-or-ganisationnelles.
- Pathologies cardiaques : à partir d’outils variés (observations, mesures, questionnaires…), contraintes cardiaques évaluées à corréler aux référentiels pour connaître au mieux tâches et postes pour protéger le salarié cardiaque et contribuer à réduire les risques pour lui et ses collègues. Pathologies chroniques, articulaires, dermatologiques, psychiatriques : exemples validés d’adaptations de poste allant du maintien au poste, aménagement, aides techniques, changement de poste, reclassement dans ou hors entreprise.

## Discussion-Conclusion

les études de postes et évaluation de risques professionnels contribuent à renforcer bonnes pratiques professionnelles et référentiels au bénéfice des travailleurs, de leur entourage et de l’environnement, d’autant que la mutualisation de données peut apporter gain de temps et compenser l’absence d’outils de métrologie.

# Research article: La couverture vaccinale contre le virus de l'hépatite B chez les professionnels de santé d'une structure hospitalière de l'est Algérien

**Published:** 2024-06-07

**Authors:** Nassima DJAHMI, Samia MÊLAIS, Zahira BOUDIAF, Hasna SEHAB, Amel BOUARICHA, Sabrina AMIRI, Adnane OTMANE, Amina BENALI, Aymen Ahmed BENTORKI, Sabrina NEDJAI

**Affiliations:** Faculté de médecine, BP205 rue Zaafrania 23000, Annaba, Algérie

## Introduction

La vaccination contre le virus de l’hépatite B est obligatoire en Algérie pour les professionnels de santé depuis l’année 2000. Au niveau de notre hôpital, la couverture vaccinale contre l’HVB du personnel hospitalier avoisine les 70 %. Du fait de l’existence de plusieurs facteurs de moins bonne réponse à la vaccination, une évaluation de l’état d’immunisation s’impose en particulier pour le personnel à risque.

## Objectif

Évaluer l’état d’immunisation contre l’HVB des professionnels de santé

## Matériel et méthodes

Il s’agit d’une étude descriptive transversale, durant l’année 2019, ayant intéressé le personnel médical et paramédical du CHU Ibn Rochd Annaba correctement vacciné contre le virus de l’hépatite B. Des prélèvements sanguins ont été réalisés par le service de la médecine du travail et adressés au service de microbiologie pour le dosage quantitatif des AC anti-Hbs. Les données ont été recueillies à partir du registre de données du laboratoire de microbiologie et les fiches de renseignement envoyées par le service de la médecine du travail.

## Résultats

337 travailleurs ont bénéficié d’un dosage des AC anti-Hbs. Il s’agit d’une population relativement jeune, l’âge moyen de la population a été de 39 ans, avec des extrêmes d’âge allant de 20 à 78 ans et une prédominance féminine de 71 %. Le taux d’immunisation était de 95 %, une proportion de 67 % était fortement immunisée. Les AC anti Hbs étaient négatifs chez 5 % des travailleurs vaccinés.

## Conclusion

L’évaluation de l’état d’immunisation de la population étudiée révèle la nécessité de mettre en place une stratégie de surveillance pour le personnel à risque.

# Research article: Stress des médecins dans les hôpitaux publics : facteurs de risques et compétences professionnelles à Yaoundé (Cameroun)

**Published:** 2024-06-07

**Authors:** Jeanne Dorothée MAA MAWO

**Affiliations:** B.P. 1280 - Institut Supérieur de Management Public (ISMP), Yaoundé

Notre travail s’est fondé sur une approche hypothético-déductive, avec une méthode de recherche quantitative. La population cible était l’ensemble du personnel médical des hôpitaux publics du Cameroun, qui acceptait de se soumettre à l’exercice de remplissage du questionnaire durant la période de l’enquête (11 - 18 septembre 2021) et notre échantillon a été de 81 médecins. L’interrogatoire reposait principalement sur les questionnaires de Robert Karasek et de Johannes Siegrist, reconnus pour aider à déterminer les facteurs de risque psycho-sociaux de stress au travail. En outre, des composantes liées à la catégorisation socio-professionnelle et aux compétences professionnelles médicales négativement influençables par le stress ont aussi fait partie intégrante du questionnaire qui a été auto-administré en ligne grâce à l’Application Google Forms. Suite à la collecte, une analyse statistique a été réalisée grâce aux logiciels SPSS version 10.0 pour Windows, R et Excel.

Il ressort de ce travail que le stress professionnel est effectivement présent chez le personnel médical du milieu hospitalier public au Cameroun. En effet, le questionnaire de Robert Karasek révèle une exposition au stress tributaire du « *Job Strain* » (forte demande psychologique et faible latitude décisionnelle) à 62,96 % et de l’« *Isostrain* » (forte demande psychologique, faible latitude décisionnelle et faible soutien social de la hiérarchie et des collègues) à 62,96 %. Par ailleurs, le questionnaire de Johannes Siegrist met en lumière une exposition au stress lié au déséquilibre efforts/récompenses à 71,60 % et au surinvestissement à 39,51 %.

Pour ce qui est des conséquences potentielles du stress au travail sur les compétences professionnelles médicales, elles sont multiples et surviennent à des degrés divers sur les compétences cognitivo-techniques et les compétences non cognitivo-techniques.

Par ailleurs, à l’issue des tests de causalité, une hypothèse a été confirmée, à savoir celle selon laquelle le stress résultant du déséquilibre efforts/récompenses des médecins aurait des effets nocifs sur leurs compétences non cognitivo-techniques.

Notons que des recommandations ont été adressées tour à tour à l’endroit du ministre de la Santé Publique, des responsables des formations sanitaires publiques et des médecins exerçant dans les hôpitaux publics, afin d’agir plus concrètement sur la prévention et la gestion du stress professionnel du personnel médical, mais aussi sur la promotion de la santé mentale au travail de ce personnel qui constitue une cheville ouvrière dans le système de santé au Cameroun.

# Research article: Prévalence et facteurs associés du stress professionnel chez les travailleurs d'une société de manutention portuaire au Bénin en 2022

**Published:** 2024-06-07

**Authors:** Mênonli ADJOBIMEY, Lassissi AFFISSATH, Hinson Antoine VIKKEY

**Affiliations:** Unité de recherche et d’enseignement en santé au travail et environnement FSS, Cotonou, Bénin

## Introduction

Le stress est un problème de santé publique, qui peut être exacerbé lorsque les employés se sentent peu soutenus et n’ont pas de contrôle sur leur travail. Il peut être causé par divers facteurs dont l’environnement social et physique du travail. La présente étude a eu pour objectif de déterminer la prévalence et les facteurs associés au stress professionnel (SP) dans une société de manutention portuaire au Bénin.

## Méthodes

Il s’est agi d’une étude transversale, descriptive et analytique menée du 29 novembre au 29 décembre 2022. Un échantillonnage non probabiliste a été effectué. Les données ont été recueillies au moyen des questionnaires de Karasek et de Siegrist. L’analyse des données a été réalisée à l’aide du logiciel R 4.1.1. Une analyse descriptive par calcul des moyennes et des proportions, suivie d’une analyse bivariée avec le test de Chi2 et calcul des Odds-Ratio bruts (OR_b_) ont été effectuées. Le seuil de significativité choisi était de 5 % (p < 0,05).

## Résultats

Au total, 656 travailleurs ont été inclus dans l’étude dont 619 (94,36 %) étaient des hommes. La prévalence du SP était de 64,18 % (IC_95_ % . [60,36 - 67,83]). Les facteurs associés au SP en analyse bivariée étaient : statut de travailleur occasionnel (OR_b_ = 1,48 IC_95_ % [1,03-2,10]); catégorie professionnelle d’agent d’exécution (OR_b_ = 5,06 IC_95_ % [2,17 - 13,2]); niveau d’étude primaire (OR_b_ = 3,91, IC_95_ % [2,34 - 6,57]); type de contrat de travail en CDD (OR_b_ = 1,87 IC_95_ % [1,34 - 2,61]); nombre d’heures moyen de travail > 8h/jour (OR_b_ = 1,88 IC_95_ % [1,22 - 2,99]).

## Conclusion

Le SP représente un risque psychologique majeur dans ce secteur d’activité nécessitant la mise en place de mesures préventives.

# Research article: Morsures de serpent : expérience du Centre hospitalier provincial de Berkane, Région Orientale du Maroc entre 2014 et 2022

**Published:** 2024-06-07

**Authors:** Zoheir TEKNI, Fouad CHAFIQ, Abdelmajid SOULAYMANI, Mohamed FEKHAOUI, Abdelrhan MOKHTARI, Rachida SOULAYMANI-BENCHEIKH

**Affiliations:** Service de réanimation - Centre hospitalier provincial de Berkane, Berkane, Maroc

## Objectif

Décrire les caractéristiques épidémiologiques cliniques et évolutives des morsures de serpent admises au Centre hospitalier provincial de Berkane située dans la région orientale du Maroc.

## Matériels et méthodes

C’est une étude rétrospective de neuf ans allant du 1^er^ janvier 2014 au 31 décembre 2022 incluant tous les cas de morsure de serpent admis au Centre hospitalier provincial de Berkane de la région orientale du Maroc.

## Résultat

Au cours de cette période, 31 cas de morsure ont été colligés avec une moyenne annuelle de 3,44 morsures par an. L’âge moyen était de 30,9 ans avec des extrêmes allant de 2 à 75 ans. Le sex-ratio était de 5,2 en faveur du sexe masculin. L’année 2016 a enregistré le pic du nombre d’admission avec sept cas. Les morsures sont survenues principalement au mois de juillet (35,5 %). Le serpent agresseur a été identifié dans deux cas : Il s’agissait *Daboia mauritanica.* Le siège de la morsure était le membre supérieur dans 35.48 % (n = 11), le membre inférieur dans 61.3 % (n = 19), le visage dans un seul cas (3.2 %). Tous les patients ont été admis pour syndrome vipérin. Le grade 0 (morsure blanche) représentait 32,2 % (n = 10), le grade 1 (envenimation mineure) était de 38,70 % (n = 12), le grade 2 (envenimation modérée) représentait 22,58 % (n = 7) et le grade 3 (envenimation grave) représentait 6.45 % (n = 2). La thrombopénie était présente chez huit patients soit dans 25,80 %. Treize patients ont bénéficié de l’antivenin Inoserp® MENA produit par Inosan Biopharm (Mexique) à raison de deux ampoules par patients en moyenne. Aucun effet indésirable immédiat n’a été enregistré. L’évolution a été favorable chez tous les patients sans séquelles. La durée d’hospitalisation moyenne était de quatre jours.

## Conclusion

Ces données montrent que la province de Berkane, de l’Oriental est une zone géographique potentiellement à risque pour la survenue d’accidents de morsure, et que le CAPM devrait renforcer sa stratégie de lutte au niveau de cette région.

# Research article: Première description de l'infection à *Rickettsia sibirica mongolitimonae* en Algérie, à Batna

**Published:** 2024-06-07

**Authors:** K. MOKRANI, P.E. FOURNIER, H. HADJ AISSA, A. BENYAHIA, R. AÏT HAMOUDA, S. TEBBAL

**Affiliations:** Service des maladies infectieuses et faculté de médecine, Batna, Algérie

## Introduction

Les énormes progrès réalisés dans le domaine de la rickettsiologie ont permis la description de nombreuses rickettsioses dites émergentes, parmi celles-ci l’infection à *Rickettsia sibirica mongolitimonae.* Plusieurs cas de la maladie ont été décrits dans le monde. Nous rapportons ici le premier cas d’infection par cette rickettsie en Algérie.

## Observation

Il s’agit d’un homme âgé de 53 ans, hospitalisé au service des maladies infectieuses de Batna en septembre 2016 pour une éruption fébrile généralisée à tout le corps épargnant le visage. L’examen physique met en évidence une tache noire au niveau de l’aisselle gauche associée à une adénopathie. Au 3^e^ jour d’hospitalisation on assiste à l’apparition d’un purpura pétéchial au niveau des deux membres inférieurs.

Le diagnostic de fièvre boutonneuse méditerranéenne est retenu et le malade mis sous doxycycline.

Une biopsie cutanée de l’escarre est réalisée et la PCR conclue à une infection a *Rickettsia sibirica mongolitimonae.*

## Conclusion

Plusieurs rickettsioses transmises par les tiques ont été décrites à travers le monde ces dernières années. En Algérie en plus de la fièvre boutonneuse méditerranéenne la plus anciennement connue, d’autres rickettsioses ont été rapportées et les rickettsies en cause ont pu être isolées de certains vecteurs. D’autres travaux associant cliniciens, microbiologistes, entomologistes et épidémiologistes viendront certainement enrichir la liste de ces rickettsioses sous nos climats.

# Research article: Coinfection tuberculose/sida : étude épidémio-clinique et thérapeutique à propos de 60 cas

**Published:** 2024-06-07

**Authors:** Z. BOUDIAF, R. MEHAMDIA, D. TLIBA, A. TOUAREF, N. DJAHMI, A. MAMMERI, S. NEDJAI, M. AIDAOUI

**Affiliations:** Hôpital Dorban - CHU Annaba, Annaba, Algérie

## Contexte

La coinfection tuberculose/VIH est une association fréquente responsable d’une grande morbi-mortalité. En Algérie, la tuberculose demeure fréquente et souvent révélatrice de l’infection à VIH.

## Objectifs

(i) mesurer la prévalence de la tuberculose chez les PVVIH suivis; (ii) déterminer le profil épidémio-clinique des patients

## Matériels et méthodes

Étude rétrospective descriptive épidémio-clinique et thérapeutique réalisée à partir des dossiers de malades coïnfectés par le VIH et la tuberculose pris en charge au CDR d’Annaba du 1^er^ janvier 1993 au 31 décembre 2022.

## Résultats

Durant la période de l’étude, 620 patients infectés par le VIH ont été colligés. La tuberculose, toute forme confondue, a été observée chez 60 patients soit une prévalence de 9,7 %.

Une prédominance masculine est notée avec un sex-ratio à 3. La localisation est dans 53,3 % cas pulmonaire, elle est extra pulmonaire (TEP) 43,3 % des cas et multiviscérale dans 3,4 % des cas.

Sur le plan clinique, les signes les plus observés sont : une toux chez 18 patients, une polyadénopathie chez 13 patients et une dyspnée chez neuf patients. Les principaux aspects radiologiques observés sont : le syndrome interstitiel dans 35 % des cas, l’épanchement pleural 18,3 % et la miliaire dans 15 % des cas.

La recherche de BAAR est positive dans 16,6 % des cas à l’examen direct et dans 35 % des cas à la culture. Le diagnostic de la TEP est établi sur la présence d’un contexte clinique évocateur avec un examen cytobactériologique du LCR ou anatomopathologique d’un prélèvement ganglionnaire ou pleural en faveur. Le taux de CD4 est réalisé chez 48 patients. Il est inférieur à 500 éléments/mm^3^ dans 2/3 des cas. Tous les patients ont bénéficié d’un traitement antituberculeux.

Le traitement antirétroviral est différé de deux semaines au moins, pour prévenir le syndrome de restauration immunitaire. Le taux de mortalité chez nos patients est de 30 %. Le décès est survenu chez neuf patients, dans les deux semaines suivant l’hospitalisation. La résistance au traitement anti tuberculeux a été observée chez un patient.

## Conclusion

le traitement ARV réduit la tuberculose active de plus de 90 % d’où l’intérêt de proposer systématiquement le dépistage du VIH chez toute personne atteinte de tuberculose et de rechercher la tuberculose chez toute personne infectée par le VIH.

# Research article: Expérience du CDR d'Annaba dans la prise en charge des migrants infectés par le VIH

**Published:** 2024-06-07

**Authors:** Z. BOUDIAF, R. MEHAMDIA, A. TOUAREF, N. DJAHMI, A. MAMMERI, S. NEDJAI, M. AIDAOUI

**Affiliations:** Hôpital Dorban - CHU Annaba, Annaba, Algérie

## Contexte

La grande majorité des migrants qui intègre le territoire algérien ignore l’existence des centres de dépistage et de prise en charge de l’infection à VIH (CDR) et surtout l’accès universel au traitement en Algérie.

## Objectifs

Déterminer les caractéristiques épidémio-cliniques des migrants pris en charge au niveau du CDR d’Annaba.

## Matériel et méthodes

Étude rétrospective et descriptive menée à partir des dossiers médicaux des patients hospitalisés et suivis à la consultation sur une période allant de 1988 au 31 décembre 2017.

## Résultats

Durant cette période 410 personnes infectées par le VIH ont été prises en charge dans notre CDR dont 30 migrants, soit 7,31 % de notre cohorte. Les personnes dépistées positives sont originaires de deux pays dans environ 90 % des cas : le Mali dans 53.33 % des cas (n = 16) et le Cameroun dans 33.33 % des cas (n = 10).

La moyenne d’âge de nos patients est de 30,83 ± 6.43 [21-49]. Les femmes représentent 30 % de cette population. La transmission hétérosexuelle est rapportée dans tous les cas. La découverte de la séropositivité a eu lieu à l’occasion d’un dépistage volontaire dans 60 % des cas (n = 18), suite à une infection opportuniste dans 36,66 % des cas (n = 11) et dans le cadre d’un suivi de grossesse dans 3.33 % des cas (n = 1).

Sur le plan clinique, un tiers des patients présente au moins une infection opportuniste. La tuberculose est retrouvée dans 10 % (n = 3) des cas, la pneumocystose pulmonaire dans 6.66 % (n = 2) des cas et la cryptococcose neuroméningée dans 3,33 % des cas (n = 1). Le taux de CD4 est inférieur à 200 chez 46,66 % des patients (n = 5). Vingt-quatre malades ont reçu un traitement antirétroviral (77,41 %) dont 17 traités systématiquement, à partir de 2015, indépendamment de leur stade clinique ou immunologique. Le taux de mortalité est de 10 %. Le décès est survenu dans la première semaine suivant l’hospitalisation 2 fois sur 3. Ces patients sont constamment perdus de vue. Le suivi à une année est noté dans 26.66 % des cas. Dans seulement 10 % des cas, le suivi était régulier pendant trois ans. Conclusion : les migrants présentent de multiples facteurs de vulnérabilité notamment au VIH. C’est une population qui est confrontée à plusieurs obstacles à l’accès aux soins. L’intervention des ONG est indispensable pour assurer, grâce à des relais communautaires, une action d’information et de sensibilisation de proximité pour cette population.

# Research article: Dépistage de l'infection à VIH chez les femmes enceintes dans les PMI d'Annaba, Algérie

**Published:** 2024-06-07

**Authors:** Z. Boudiaf, R. Mehamdia, A. Touaref, N. Djahmi, A. Mammeri, N. Messalhi, S. Nedjai, M. Aidaoui

**Affiliations:** Hôpital Dorban - CHU Annaba, Annaba, Algérie

## Contexte

Le risque de transmission du VIH de la mère au fœtus est considérable si la mère ne bénéficie pas d’un traitement antirétroviral. En Algérie, la prévalence nationale du VIH/sida parmi les consultantes prénatales est de 0,09 %, cependant le taux du dépistage de l’infection à VIH dans cette population reste faible. Le dépistage précoce du VIH chez la femme enceinte (FE) et la prise en charge adaptée des séropositives permet de réduire le risque de transmission verticale du VIH.

## Objectifs

(i) mesurer la séroprévalence de l’infection à VIH chez les femmes enceintes dans la région d’Annaba; (ii) déterminer le profil sociodémographique de la population étudiée.

## Matériel et méthodes

Étude transversale descriptive, avec consentement éclairé, menée au niveau de quatre centres de protection maternelle et infantile (PMI) de l’Établissement public de santé de proximité (EPSP), durant l’année 2016 sur un échantillon représentatif des femmes enceintes. Le test rapide à orientation diagnostique (TROD) « KASHIF » a été utilisé pour le dépistage de l’infection à VIH.

La confirmation des tests positifs se fait selon l’algorithme national du diagnostic biologique du VIH.

## Résultats

Durant la période de notre étude, 1 019 femmes enceintes ont été dépistées pour le VIH au niveau des PMI retenues parmi 1 762 sensibilisées, soit un taux d’acceptabilité de 57,83 %.

La moyenne d’âge est de 30,11 ans ± 5.4; la tranche d’âge la plus représentée est celle de [25-29 ans] avec un taux de 32 %. Parmi les tests réalisés, quatre tests sont positifs soit une séroprévalence de 0,39 %. Toutes les femmes enceintes dépistées positives pour le VIH sont mariées, appartenant à la tranche d’âge 25-29 ans dans la moitié des cas et ont un niveau d’instruction moyen ou secondaire.

## Conclusion

Notre étude montre que l’infection à VIH est une réalité chez les femmes enceintes. Le taux de séroprévalence retrouvé est élevé par rapport aux taux nationaux. Le dépistage de l’infection à VIH conditionne l’accès de la femme enceinte séropositive à une prise en charge précoce et adaptée pour éliminer le risque de transmission maternofœtale (TME). Des directives nationales recommandent de proposer systématiquement le dépistage du VIH pour toute femme enceinte, cependant, leur application reste limitée.

# Research article: Analyse des mécanismes de coordination des interventions de soins et soutien en faveur des orphelins et enfants rendus vulnérables (OEV) du fait du VIH/sida et leurs familles

**Published:** 2024-06-07

**Authors:** Solange AMETHIER, Baba SANGARE, Mariette Alida Christine BAKOU NIANGORAN

**Affiliations:** Cellule médico-sociale du ministère de la Femme, de la Famille et de l’Enfant, Abidjan, Côte d’ivoire

Le ministère de la Femme, de la Famille et de l’Enfant, à travers sa Cellule médico-sociale, a mené une étude sur l’analyse des mécanismes de coordination des interventions de soins et soutien en faveur des orphelins et enfants redus vulnérables (OEV) du fait du VIH/sida et leurs familles. Cette étude a consisté à examiner les mécanismes de coordination des interventions de prise en charge des OEV et de leurs familles à travers la recherche documentaire et l’administration d’un guide d’entretien. Ainsi, ont participé à l’étude, 20 plateformes de collaboration OEV, 8 programmes nationaux et 7 partenaires techniques et financiers intervenant dans l’offre de services en faveurs des OEV et leurs familles. Les résultats de ce travail de recherche révèlent la nécessité d’une réponse multisectorielle basée sur une complémentarité des interventions des acteurs, le renforcement du suivi des activités et du cadre de concertation, le partage d’informations et le développement des stratégies de pérennisation des interventions. Tout ceci dans un contexte où le sida représente un problème de Santé Publique, car il n’existe pas jusqu’à ce jour un traitement curatif pour enrayer cette pandémie qui touche la frange de la population la plus vulnérable y compris les enfants. La prise en compte des résultats issus de l’étude par les acteurs impliqués dans l’offre de services en faveurs des OEV et leurs familles contribuera à l’amélioration de la prise en charge de ces enfants et au renforcement du mécanisme de coordination des interventions de soins et soutien dans un environnement stable.

# Research article: Dépression et risque suicidaire chez les adultes infectés par le VIH au CHU de Brazzaville : prévalence et facteurs associés.

**Published:** 2024-06-07

**Authors:** Rolland Bienvenu OSSIBI IBARA, A. MOUKOUMA, T. DOUKAGA, M. EKAT, P. BINTSINDOU, F. KINGA, E. ANGONGA, P. BENDETT, A.-M. MOUANGA

**Affiliations:** Université Marien Ngouabi, Brazzaville, Congo

## Objectif

Déterminer la prévalence de la dépression associée au risque suicidaire (RS) et identifier les facteurs associés

## Patients et méthode

Il s’est agi d’une étude prospective à visée descriptive et analytique allant du 1^er^ janvier au 30 novembre 2022 et incluant tous les patients VIH positif recevant ou non un traitement antirétroviral. Le questionnaire patient Heath Questionnaire 9 (PHQ-9) a servi pour évaluer le degré de dépression et du risque suicidaire. L’observance thérapeutique a été évaluée au moyen du questionnaire de Morisky. Les données ont été analysées au moyen du logiciel SPSS version 23. Les variables qualitatives ont été exprimées en effectifs et pourcentage et celles quantitatives en moyennes et écart-type. Les tests statistiques ont été utilisés selon leurs critères d’applicabilité. Pour tous les tests, le seuil de significativité a été fixé à 0,05.

## Résultats

La prévalence de la dépression et du risque suicidaire était de 32 %. Il s’agissait des patients d’âge moyen de 42,83 ± 10,24 ans, de sexe féminin (n = 101; 67,3 %), chrétiens (n = 97; 64 %), célibataires (45,3 %). La stigmatisation (50 %) était le principal motif de tentative de suicide. Le VIH était perçu comme traitable (68,7 %), malchance (n = 57; 41,3 %). Les patients étaient au stade 1 de l’OMS (n = 105; 70 %). Le risque de suicide était présent dans 47 cas (31,3 %). Les facteurs associés à la dépression et au risque suicidaire étaient l’âge (p = 0,000), la résidence (p = 0,028), la tentative de suicide (p = 0,000), le désir de procréation (p = 0,000) et les effets secondaires des ARV (p = 0,000).

## Conclusion

la prévalence de la dépression et le risque suicidaire était élevée et ce en lien avec la stigmatisation et les conditions socio-économiques. Les facteurs associés étaient en rapport avec ceux identifiés dans la littérature. Il est nécessaire d’intégrer la santé mentale dans la prise en charge globale des PvVIH.

# Research article: Vécu psychologique des femmes vivant avec le VIH à Brazzaville en 2022

**Published:** 2024-06-07

**Authors:** Rolland Bienvenu OSSIBI IBARA, G. OBEMBO, P. M. BINTSINDOU, T. ADOUA DOUKAGA, M. EKAT, E. ANGONGA PABOTA, P. BENDENT, F. KINGA, C. ITOUA

**Affiliations:** Université Marien Ngouabi, Brazzaville, Congo

## Objectif

Évaluer le vécu psychologique des femmes vivant avec le VIH au CHU de Brazzaville

## Patients et méthode

Étude transversale incluant les femmes âgées d’au moins 18 ans, infectées par le VIH durant la période allant du 1^er^ avril au 30 septembre 2022 (6 mois) sans antécédents psychiatriques et consentants à l’étude.

## Résultats

Quatre-vingts patientes âgées en moyenne de 45,92 ± 14,5 (21-69) ans, célibataires (n = 33; 41,3 %), commerçantes (n = 30; 37,5 %), résidant en ville (n = 48,7 %). Le niveau socio-économique était bas (n = 30; 37,5 %). La durée moyenne de l’infection à VIH était de 118,7 mois. Le counseling prétest n’avait pas été fait (n = 66; 82,5 %) et le VIH avait été découvert lors d’un bilan de santé (n = 47; 58,8 %). La distance domicilesite de dispensation des ARV était comprise entre 6-10 km dans 51,3 %. Les patientes étaient sous protocole TDF+3TC+DTG (n = 35; 43,7 %). Le statut sérologique du partenaire n’était pas connu (n = 41; 51,3 %) et le désir d’avoir un enfant était exprimé (n = 21; 77,8 %). Le statut sérologique était divulgué dans la famille (n = 50; 62,5 %). La tuberculose était la première infection opportuniste retrouvée (n = 15; 42,8 %) et les patientes étaient au stade 4 de l’OMS (n = 43; 52,5 %). Les troubles psychologiques étaient l’anxiété (n = 32; 40 %) et la dépression modérée (n = 9; 3,7 %). L’estime de soi était très faible (n = 26; 32,5 %) et faible (n = 37; 46,3 %). Les facteurs associés étaient : l’âge (p = 0,03), le niveau d’instruction (p = 0,02), la résidence (p = 0,02) de même que le niveau socio-économique (p = 0,02), le counseling pré-test non fait (p = 0,003).

## Conclusion

La perte de l’estime de soi, l’anxiété et la dépression sont fréquentes chez les femmes vivant avec le VIH à Brazzaville et ce en lien avec la stigmatisation, le bas niveau socio-économique et l’absence d’information claire sur le VIH. Il est donc important de prendre en compte la santé mentale dans la prise en charge globale des PvVIH.

# Research article: Donner espoir d'une vie en couple aux PvVIH sérodifferents : expérience des assistants psycho sociaux du Centre de Traitement Ambulatoire (CTA) de l'Hôpital National Donka, en Guinée

**Published:** 2024-06-07

**Authors:** Bah Aïssatou LAMARANA, Joseph BALATH, Aïssatou Lamarana Bailo DIALLO, Brigitte VAKOU SINGA, Mamadou Djoulde KANTÉ, Mohamed CISSE, Thierno Mamadou TOUNKARA

**Affiliations:** Fondation Espoir Guinée, Hôpital Donka, Conakry, Guinée

## Introduction

La découverte de la séropositivité au VIH est source de stigmatisation de la part de la communauté ce qui rend davantage difficile toute possibilité de s’engager dans une vie de couple séronégatif. Aujourd’hui grâce aux traitements et à une bonne hygiène de vie, une personne porteuse du VIH peut avoir une vie familiale avec une personne séronégative. A travers le monde, jusqu’à 50 % des personnes séropositives qui entretiennent des relations durables ont des partenaires séronégatifs, selon l’OMS.

## Matériel et méthodes

Nous avons réalisé une étude monocentrique, longitudinale, à visée descriptive et analytique dans le centre de traitement ambulatoire des personnes vivants avec le VIH au service de dermatologie vénérologie du CHU Donka. L’étude s’est déroulée sur une période de trois ans allant du 1^er^ janvier 2020 au 31 décembre 2022.

## Résultats

Nous avons inclus 102 patients demandeurs en mariage enregistrés, puis célébré 67 mariages dont 56,71 % était de sexe féminin avec une moyenne d’âge de 30 ans [18-50] et 32 couples ont eu des enfants, 3 femmes en état de famille, 35 de nos patients vivent ensemble et sont à la recherche d’enfants. Et 35 autres sont à la recherche d’un partenaire.

## Conclusion

La vie en couple sérodifférent reste une question d’actualité et nécessite un appui sanitaire, psychique et social. La nouvelle campagne de prévention sida devrait porter un accent sur le partage de la vie avec une personne séropositive.

# Research article: Connaissances, attitudes et pratiques des ménages du district sanitaire de Cocody-Bingerville sur les mesures de prévention et de contrôle de la dengue, Côte d'Ivoire

**Published:** 2024-06-07

**Authors:** Marie Noëlle ANO AMA KOUNANGUI, Daniel Ekra KOUADIO, Franck Kokora EKOU, Maky DIALLO, Konan N’GUESSAN, Joseph Bénie BI VROH, Simplice Dagnan N’CHO

**Affiliations:** Université Félix Houphouët Boigny, Abidjan, Côte d’Ivoire

## Introduction

La Côte d’Ivoire a connu successivement trois épidémies de dengue, avec toujours le district sanitaire de Cocody-Bingerville comme district de départ.

## Objectif

Déterminer les facteurs favorisant la recrudescence des épidémies de dengue dans le district sanitaire de Cocody-Bingerville.

## Méthodes

Il s’agit d’une étude prospective menée dans trois aires de santé du district sanitaire de Cocody-Bingerville. La technique par choix raisonné a été utilisée pour la répartition des quartiers constituant le district.

## Résultats

Au total, 383 ménages ont été interrogés dans les aires de santé de Cocody-Centre, Deux-Plateaux et Angré avec respectivement 33,94, 33,15 et 32,91 % des répondants. Le sex-ratio était de 1,2 et l’âge moyen entre 28 et 38 ans. Avec 77,89 % de répondants qui avaient déjà entendu parler de la dengue et 40,73 % des sujets connaissaient le moustique tigre comme vecteur de la dengue. Dont 85,63 % des cas affirmaient que le vecteur *(Aedes)* se développe dans les flaques d’eau, 72,06 % parlaient d’eau stagnante, 57,96 % des ordures et 10,72 % mentionnent des pots de fleurs. Sur les moyens de prévention contre la dengue, 66,05 % des enquêtés citaient la bonne gestion des eaux usées, 62,66 % les moustiquaires, 59 % l’assainissement de la ville, 58,22 % l’utilisation des MILDA et 20,42 % des enquêtés ne connaissaient pas les moyens de prévention contre la dengue. Plus de la moitié de la population enquêtée (52,22 %) des cas stockaient les déchets à l’intérieur des maisons. Aussi, 47,78 % des répondants ont évacué les ordures ménagères par le pré-collecteur au-delà de trois jours. Les répondants (64,50 %) exposaient de vieux objets à la pluie (canaris, casserole, pneus, carcasse de véhicule, noix de coco).

## Conclusion

Cette étude révèle le manque de connaissances sur les mesures préventives contre la dengue et le mode de vie de la population favorable à la survenue des épidémies du district sanitaire de Cocody-Bingerville.

# Research article: De la déclaration d'Alma Ata à celle de Montréal, l'intelligence artificielle va t'elle emprunter les sentiers battus par les soins de santé primaires : une analyse éthique de la question sous le prisme de la pandémie à Covid-19 et données personnelles

**Published:** 2024-06-07

**Authors:** Ousmane LY, Abdoulaye KONATÉ, Souleymane Sawadogo, Hammadoun DIA

**Affiliations:** Faculté de médecine et d’odonto-stomatologie (FMOS), Université des sciences, techniques et technologies de Bamako (USTTB) - Agence nationale de télésanté et d’Iinformatique médicale (ANTIM) - Bamako

Les questions éthiques liées à l’intelligence artificielle, aux soins de santé primaires, à la pandémie de Covid-19, à la collecte de données personnelles, aux lois et réglementations et aux déclarations internationales relèvent d’une importance capitale et d’une actualité poignante. L’intelligence artificielle est le domaine de l’informatique qui vise à créer des machines capables de reproduire certaines capacités humaines. Les soins de santé primaires sont décrits comme la première étape pour traiter les problèmes de santé et sont considérés comme une partie essentielle de tout système de santé efficace et accessible. La pandémie de Covid-19 a entraîné des mesures de confinement et de distanciation sociale sans précédent, affectant profondément les économies et les sociétés du monde entier dont les questions des données. La collecte de données personnelles est gouvernée par des lois et des réglementations spécifiques, telles que le RGPD en Europe. Enfin, les déclarations internationales sont des expressions formelles d’engagements ou de principes partagés par plusieurs pays ou organisations à travers le monde, pouvant contribuer à établir des normes communes pour les politiques et les pratiques dans un domaine spécifique. La déclaration de Montréal devrait suivre le même chemin que celle d’Alma Ata et des droits humains. Ce qui permettrait l’application de principes éthiques pertinents inspirés du modèle « principaliste » de Beauchamp et Childress que sont la transparence, la responsabilité, la non-discrimination et la protection de la vie privée. Bien que ce modèle ait été critiqué pour ne pas tenir compte de la culture, de la diversité et des contextes sociaux, il reste un cadre important pour l’éthique médicale, applicables aux nouveaux défis.

Le cheminement à travers le prisme de la pandémie à Covid-19 et données personnelles, nous a poussé à penser que la déclaration de Montréal doit tirer profit des expériences mondiales avec la bonification de la dynamique inclusive de la co-construction pour une utilisation éthique de l’intelligence artificielle et des mégadonnées de la santé au service de notre humanité.

# Research article: PORTS - Plateforme ouverte de ressources tropicales en santé : bilan après neuf mois d'existence

**Published:** 2024-06-07

**Authors:** Jean-Marie MILLELIRI, Jean-Loup REY, Jean-Paul BOUTIN

**Affiliations:** GISPE - 82 boulevard Tellène - 13007 Marseille, France

Avec le soutien de l’Agence française du développement (AFD), le GISPE (Groupe d’intervention en santé publique et épidémiologie) a développé une interface numérique pour rendre disponible aux chercheurs et acteurs de santé des documents pas toujours facilement accessibles lors de recherche bibliographique. Cette interface dénommée PORTS pour plateforme ouverte en ressources tropicales de santé, est constituée de registres thématiques pouvant être interrogés pour retrouver les documents mis en ligne. Les documents sont des articles en open access, des photographies, des illustrations, des présentations, des thèses ou des mémoires de recherche et aussi toutes les communications données lors des Actualités du Pharo. L’accès à PORTS est gratuit sur inscription sur le site www.ports.fr. Après neuf mois d’existence plus de 300 utilisateurs sont inscrits issus de 45 pays. La base documentaire en constante évolution comprend plus de 400 documents.
